# Polysaccharides—Important Constituents of Ice-Nucleating Particles of Marine Origin

**DOI:** 10.1021/acs.est.4c08014

**Published:** 2025-03-07

**Authors:** Susan Hartmann, Roland Schrödner, Brandon T. Hassett, Markus Hartmann, Manuela van Pinxteren, Khanneh Wadinga Fomba, Frank Stratmann, Hartmut Herrmann, Mira Pöhlker, Sebastian Zeppenfeld

**Affiliations:** † Department of Atmospheric Microphysics (AMP), Leibniz Institute for Tropospheric Research (TROPOS), Leipzig 04318, Germany; ‡ Department of Modeling Atmospheric Processes (MOD), Leibniz Institute for Tropospheric Research (TROPOS), Leipzig 04318, Germany; § Department of Arctic and Marine Biology, UiT − The Arctic University of Norway, Tromsø 9019, Norway; ∥ Atmospheric Chemistry Department (ACD), Leibniz Institute for Tropospheric Research (TROPOS), Leipzig 04318, Germany

**Keywords:** ice-nucleating particles, ice-nucleating macromolecules, polysaccharides, remote marine regions

## Abstract

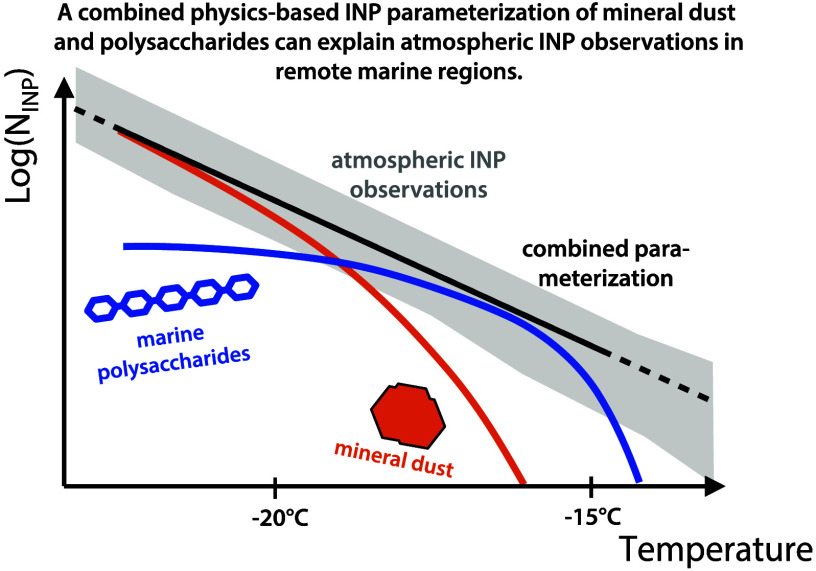

Remote marine regions are characterized by a high degree of cloud cover that greatly impacts Earth’s radiative budget. It is highly relevant for climate projections to represent the ice formation in these clouds. Therefore, it is crucial to understand the sources of ice-nucleating particles (INPs) that enable primary ice formation. Here, we report polysaccharides produced by four different aquatic eukaryotic microorganisms (*Thraustochytrium striatum*, *Tausonia pullulans*, *Naganishia diffluens*, *Penicillium chrysogenum*) as responsible ice-nucleating macromolecules (INMs) in these samples originating from the marine biosphere. By deriving a classical nucleation theory-based parametrization of these polysaccharidic INMs and applying it to global model simulations, a comparison to currently available marine atmospheric INP observations demonstrates a 44% contribution of polysaccharides to the total INPs of marine origin within −15 to −20 °C. The results highlight the relevance of biological INMs as part of the INP population in remote marine regions.

## Abstract 2

The ability of aquatic fungi and protists to form ice is caused by polysaccharides. In the remote marine atmosphere, these macromolecules can be as important as mineral dust ice-nucleating particles in the temperature range relevant for mixed-phase clouds.

## 1 Introduction

Ice-nucleating particles (INPs) initiate the formation of ice crystals in clouds and consequently impact the radiative balance of the Earth’s atmosphere and precipitation formation and are relevant for understanding climate sensitivities.^1^ For large parts of the remote oceans, particularly the Southern Ocean where INP concentrations are low,^2,3^ discrepant observations exist in the representation of cloud phase, driven by strong biases in the radiative effect variables of atmospheric models.^4^ A better understanding of INP sources, such as mineral dust or sea spray aerosol (SSA),^5−7^ INP transport and underlying mechanisms of ice nucleation at the molecular level offer a large potential to improve climate modeling, especially for the sensitive, but still quite unexplored climate hot spots, such as the Southern Ocean and the Arctic.^8−13^ In general, the ocean is known to be a source of INPs because it contains ice-active microorganisms (and parts or products thereof) that can enter the atmosphere as part of SSA.^6,14−19^ Increased biological activity of ocean water for example during a phytoplankton bloom thus provides a larger reservoir for INPs that can be aerosolized.^6,15−17,20−23^ Indications have been found that organic compounds produced from marine microorganisms may be responsible for the observed ice-nucleating ability^15−17,24^ and can be enriched in the ocean surface microlayer.^15,25−28^ Various marine microorganisms have been investigated and found to be active in nucleating ice, including bacteria,^16,29−33^ algae,^14,33−35^ marine diatoms,^29,32,33,36−39^ haloarchaea,^40^ viruses,^41^ and fungi.^31,42^ Alongside algae and bacteria as primary contributors to biomass production and degradation, marine fungi are now gaining recognition for their significant role in the carbon cycle.^43,44^ However, significant knowledge gaps remain regarding the geographical and annual distribution of fungi and pseudofungi. These organisms are underrepresented in environmental surveys due to their absence from databases,^45^ and their potential role as INPs remains largely unexplored. The latter is the subject of this study.

Ice-nucleating macromolecules (INMs) cause the activity of biogenic INPs.^46^ While the ice-nucleating activity (INA) of terrestrial microorganisms and pollen have been attributed to specific proteins or polysaccharides, respectively,^47−51^ little is known about the chemical identity of biogenic marine INMs from SSA to date. Studying planktonic microorganisms, Wolf et al.^52^ found strong evidence that proteinaceous and saccharidic components determine the INA of organics in SSA. Alpert et al.^24^ found proteins and polysaccharides in both ambient and laboratory-generated INPs containing exudates from planktonic microbes. Further, a polysaccharidic nature of marine INMs appears plausible based on correlations between INA and free glucose, a non-ice-active monosaccharide and degradation product of polysaccharides, found in the Arctic surface seawater.^53^ To identify and quantify the contribution of specific ice nucleation active components to marine INP population, targeted measurements of the chemical compounds are required.^16,17^

For high predictability modeling of atmospheric INP concentrations over remote marine regions, two main INP sources are commonly taken into account: mineral dust and marine organics derived from SSA.^54−56^ Therefore, state-of-the-art INP parametrizations are applied which either consider unspecified INP concentrations or use bulk aerosol properties as proxies such as number, surface area, or broad categories of aerosol constituents (e.g., mineral dust, sea salt).^8,57−59^ In doing so, mostly a log-linear or polynomial relationship between INP concentration and temperature is assumed.^15,57,59−62^ Although this approach generally reflects the observed temperature-dependent INP concentrations, extrapolating to a wider temperature range than supported by observations is not necessarily valid and lacks a physical basis. Furthermore, apart from mineral dust, the INP concentration is therefore usually estimated only by indirect proxies, i.e., not directly based on the presumably different chemical aerosol components that actually cause the freezing in different temperature regimes. For mineral dust, different ice-nucleating components are accounted for already.^4,63^

In this study, we investigate the ice nucleation activity of marine polysaccharides derived from marine fungi and protists as well as from commercially available standard polysaccharides. We develop a physically based parametrization and integrate these findings together with mineral dust INPs in a global model to assess their relevance by comparing with available atmospheric INP observations in marine regions.

## 2 Experimental Methods

### 2.1 Isolation and Cultivation of Microorganisms

The abundance of nonphototrophic microorganisms was determined in the marine environment by extracting and sequencing DNA collected during a cruise on the R/V Helmer Hanssen near Svalbard in November 2017 in different environmental compartments including airborne and surface water samples (Table S1, Figure S1, and details described in the Supporting Information (SI)). Microorganisms from various environments, including nearshore Arctic sediment in Tromsø and Arctic marine plankton, were sampled, focusing on heterotrophic eukaryotic microbes such as fungi and a protist. The collected samples were cultivated for further analysis (see Table S2, SI). Specifically, we studied one thraustochytrid (*Thraustochytrium striatum*), two yeasts (*Tausonia pullulans* and *Naganishia diffluens*), and one filamentous fungus (*Penicillium chrysogenum*).

### 2.2 Microphysical and Chemical Analyses

The ice nucleation activity of these cultivated fungi and thraustochytrids was analyzed using a droplet freezing assay (Section S4 SI). To constrain potential candidates for ice-nucleating macromolecules produced by these microbes different physical and chemical pretests were done including a 0.2 μm membrane filtration,^15,17,64^ heat treatment (95 °C for 1 h),^65,66^ and CaCl_2_ precipitation (1.6 g L^–1^ CaCl_2_ added to 0.2 μm filtered aliquots of *T. pullulans*). To confirm the preliminary observations in the microbial samples, the following commercially available standard polysaccharides were also analyzed: laminarin, λ-carrageenan, κ-carrageenan, alginic acid (long- and short-chained), agar, cellulose, xanthan gum, and a lipopolysaccharide from *P. aeruginosa*. The total combined carbohydrates (TCCHO, i.e., hydrolyzable polysaccharide) content and monosaccharide composition was quantified after an acid hydrolysis (0.8 M HCl, 100 °C, 20 h) using a high-performance anion-exchange chromatography with pulsed amperometric detection^67^ as described in detail in the SI. For the determination of total organic carbon (TOC), 20 μL of the liquid culture samples were pipetted on a rectangular punch (1.5 cm^2^) of quartz fiber filter, dried up for 20 min at room temperature, and analyzed on the Lab OC-EC analyzer (Sunset Laboratory Inc., USA) applying the standard temperature protocol EUSAAR2.91.

### 2.3 Application of INP Parametrization to Global Model Simulations

Existing simulation data for the year 2010^68^ that was generated with the atmospheric chemistry transport model TM5^69^ was used in the present study. The data set was chosen as it provides the necessary proxy mineral dust and sea salt for calculating INP concentrations and a high time resolution (hourly). From the simulation, the mass concentration of sea salt and mineral dust as well as the number concentrations in the soluble and insoluble accumulation and coarse modes were used to derive INP concentrations. For INPs from mineral dust, the parametrization by Niedermeier et al. (2015, N15)^70^ was used. INPs from marine polysaccharides are calculated by applying the parametrization derived in this work (HSZ25). A dynamic emission modeling of polysaccharides and, hence, the aerosol polysaccharide content is beyond the scope of this study. Therefore, it is assumed that a constant percentage of 0.5 and 0.1% of the sea salt mass in the soluble accumulation and coarse mode, respectively, consists of marine polysaccharides. This polysaccharide fraction in SSA is derived from ambient measurements conducted during the PI-ICE campaign in the Southern Ocean (0.2–0.5% in accumulation mode, 0.03–0.05% in coarse mode, campaign mean in the respective size bins of the size-resolved impactor data),^67^ the PASCAL campaign in the central Arctic Ocean (0.1–0.9% in accumulation mode, 0.1–0.7% in coarse mode, campaign mean in the respective size bins of the size-resolved impactor data),^71^ on Cape Verde (0.1% campaign mean in PM10),^72^ and at Mt. Zeppelin, Svalbard, Norway (0.4% campaign mean in PM1).^73^ Despite the limited data basis of polysaccharide measurements, the range of single measurements as well as the mean polysaccharide content in SSA seems to be similar between the Arctic and Southern Ocean. To estimate the uncertainty caused by this assumption, a lower and upper bound for the polysaccharide content of 0.05 and 0.5%, respectively, in both accumulation and coarse mode was used as well. By applying a mass fraction, co-emission of sea salt and marine organic aerosol is directly reflected. Hence, uncertainties from modeling, estimating concentrations of organics or biological activity in the sea surface microlayer, and enrichment factors during aerosolization are avoided. On the other hand, the spatiotemporal variability of the polysaccharide concentrations in aerosol and more detailed emission descriptions cannot be easily considered at present. For comparison to our polysaccharide-based INP parametrization (HSZ25), the INP parametrization of McCluskey et al. (2018, M18)^59^ is applied, representing an estimate of nonheat-labile marine INPs in sea spray aerosol, hence, originating from seawater, and by purpose avoiding any influence of mineral dust. INP concentrations were calculated for the hourly model data and averaged over the whole year. The modeled INP concentrations were evaluated against observational data sets from remote marine regions covering more than a decade. Campaigns in the Southern Ocean provided the largest share of observations, but also the Arctic Ocean, as well as Northern and Tropical Atlantic and Pacific, are represented. By using the annual average INP concentration, the effects of the short-term variability of mineral dust concentrations in the Southern Ocean region are avoided. As both the modeled mineral dust and sea salt concentrations in the Southern Ocean do not show an obvious seasonal cycle (see also the SI), we assume that derived annual average INP concentrations in such remote marine regions are similar in different years. Further, the main conclusions are drawn from the comparison of HSZ25 and M18 that use the same proxy, i.e., the modeled sea salt concentration, hence both parametrizations would have the same deviations from the exact conditions during the individual measurement periods. The HSZ25 parametrization is evaluated against M18 in terms of improved agreement of modeled and observed INP concentrations within a factor of 10 (FAC10). This refers to the increase in FAC10 in addition to mineral dust INPs only. The fraction of improved agreement within a factor of 10 explained by polysaccharides F_HSZ25, M18_ (right column in Table S5) is calculated as
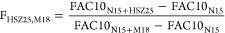
1

where FAC10_N15_ is the agreement against the observations within a factor of 10 when using only mineral dust INPs (according to N15). Accordingly, FAC10_N15+M18_ is that agreement when using the combination of N15 with marine INPs derived from sea spray aerosol according to M18, and FAC10_N15+HSZ25_ when using the combination of N15 with marine polysaccharide INPs.

### 2.4 Data and Code Availability

Sequence results of eukaryotic microorganisms are available https://www.ncbi.nlm.nih.gov with the respective accession numbers given in Table S2. Sequence abundance of airborne and aquatic microorganisms sampled in November 2017 on R/V Helmer Hanssen, and INM concentrations of different microorganisms, polysaccharides, and targeted tests can be found on 10.5281/zenodo.10421589. Data on combined carbohydrates and inorganic ions in size-resolved ambient aerosol particles during PI-ICE (Southern Ocean) and Cape Verde can be accessed at https://doi.pangaea.de/10.1594/PANGAEA.927565 and https://doi.pangaea.de/10.1594/PANGAEA.969080, respectively. The applied codes are available on 10.5281/zenodo.10423597.

## 3 Results and Discussion

### 3.1 Polysaccharides Responsible for INA of Marine Eukaryotic Microorganisms

**Figure 1:**
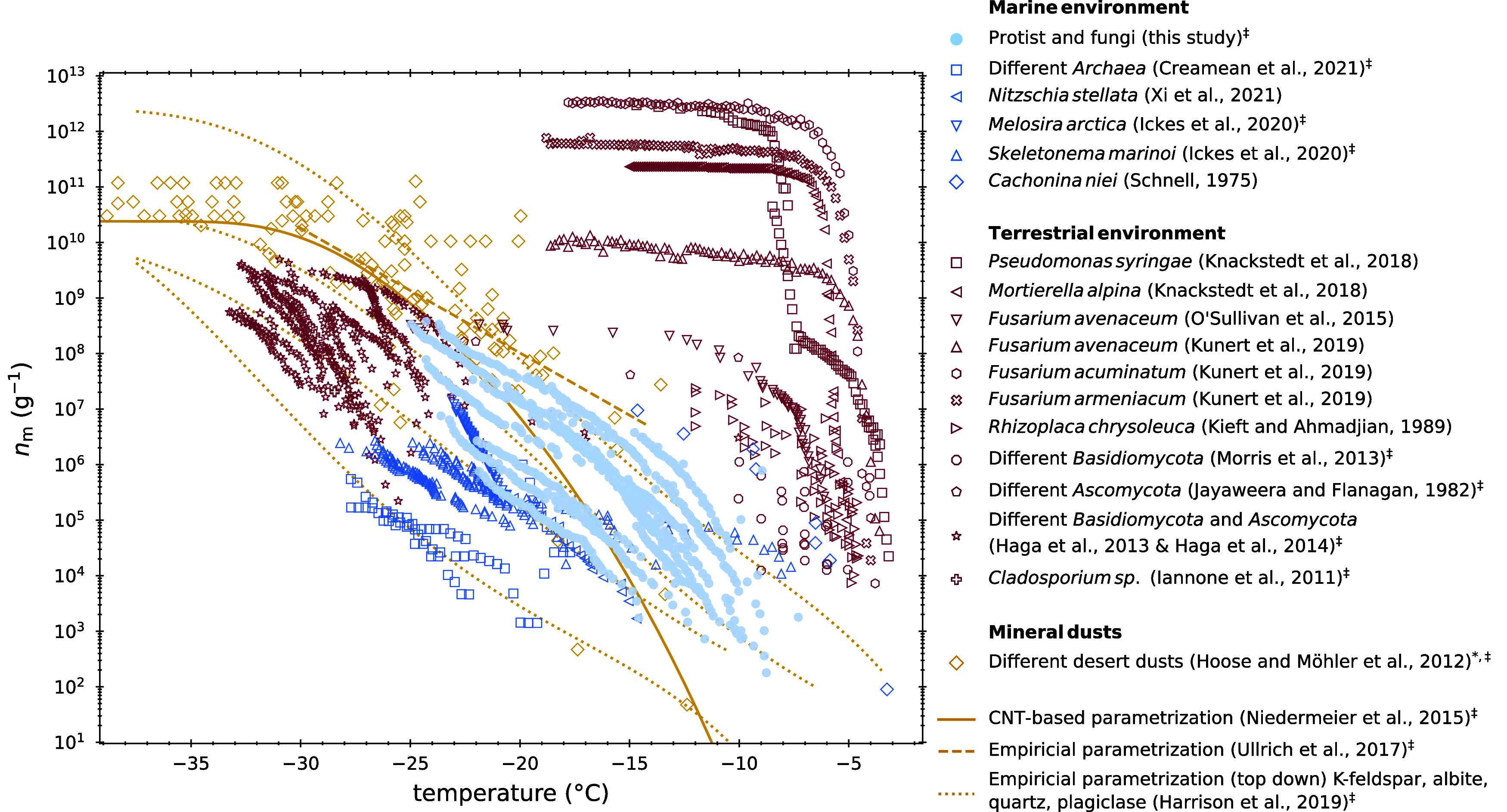
Ice nucleation site density per mass *n*_m_ is given as a function of temperature for known mineral dust, biogenic INMs, and in this study examined marine microbial INMs. ‡This data was converted from *n*_s_ or *n*_v_ to *n*_m_. Details of the conversion are given in the SI. * The desert dust compilation includes Asian dust, Canary Island dust, Israeli dust, and Saharan dust. Refs^21,38−40,50,61,62,70,74−82^

**Figure 2:**
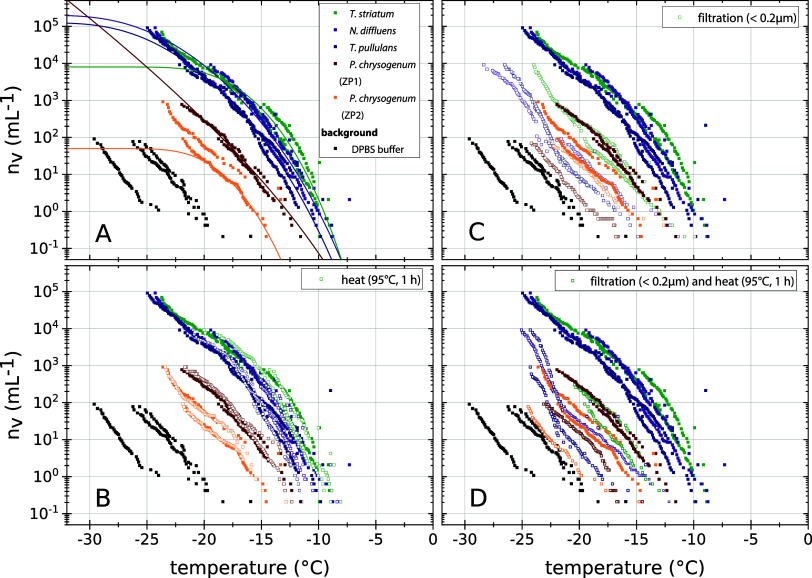
Physical properties of analyzed eukaryotic microorganisms indicate a non proteinaceous origin of INMs mainly attached to the microbial cells. The INM number concentration per sample volume is given for unmodified eukaryotic microorganisms together with CNT-based fits (A) and after physical treatments: heating at 95 °C for 1 h (B), filtration (<0.2 μm; C) and the combination of both (D). Heating does not change INM number concentrations, whereas filtration significantly reduces INM number concentration, and subsequent heating does not alter the INM concentration. This points toward the existence of heat-stable most likely non proteinaceous INMs freely suspended and to a larger extent connected to microbial cells.

**Figure 3:**
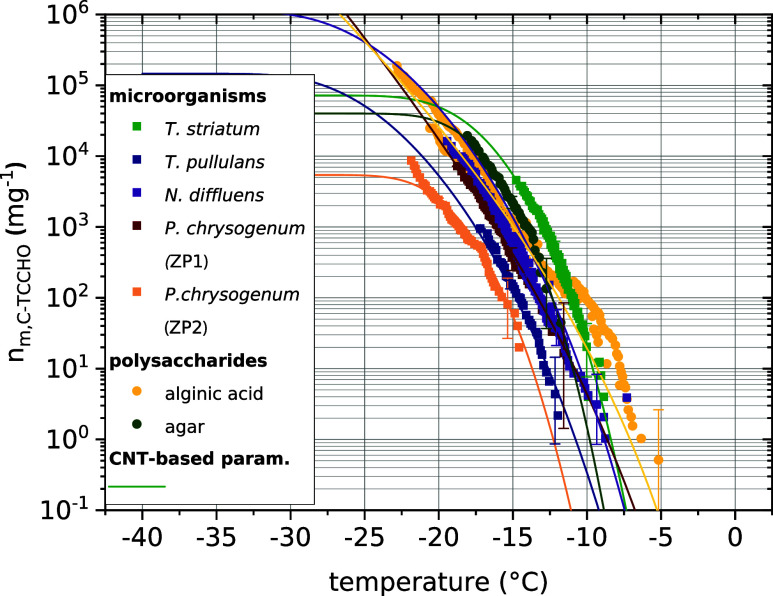
High agreement of the ice nucleation activity of tested aquatic eukaryotic microorganisms with that of marine polysaccharides. The temperature-dependent ice nucleation site densities per carbohydrate carbon mass *n*_m, C-TCCHO_ is presented with CNT-based parametrizations (parameters are presented in Table S4 in the SI). The derived CNT-parametrization of *T. striatum* is used for HSZ25.

In the Arctic marine environment, even during polar night, a sequencing-based survey of airborne marine eukaryotic microorganisms revealed an unexpected abundance of fungi (Figure S1). As a basis for the present study, we isolated and cultivated four different eukaryotic microorganisms present in the ocean and the atmosphere and studied their INA: (Figure 1) one thraustochytrid (*T. striatum*), two yeasts (*T. pullulans*, *N. diffluens*), and one filamentous fungus (*P. chrysogenum*). The marine microorganisms showed a significant spread of approximately 3 orders of magnitude at −15 °C. Compared to other INP types, these marine microbes induced freezing at higher temperatures than most types of desert dusts (above −18 °C) and at lower temperatures than (mainly proteinaceous) biogenic INMs from terrestrial origin (below −8 °C). Similar temperature dependencies among the different microorganisms indicated by a parametrization based on classical nucleation theory (CNT) (Figure 2) strongly suggest that the same chemical class of INMs is responsible for the observed INA, which was investigated in detail as described below:

(i) To determine the molecular identity of the INMs, various physical and chemical tests were conducted on the microbial cultures. Filtration through a 0.2 μm filter reduced INM concentrations by 97–99.8% (Figure 2C), though still above background levels. This indicates that INMs are predominantly attached to microbial cells or aggregates, with a smaller portion existing as dissolved or colloidal substances. Heating the suspensions (95 °C for 1 h, Figure 2B,D) did not alter INM concentrations, suggesting a nonproteinaceous nature, as proteins typically lose their ice-nucleating activity at high temperatures.^46,66,83^ In contrast, strong acid hydrolysis of the *T. pullulans* sample (0.05 M H_2_SO_4_, 100 °C, 72 h, Figure S2B) significantly reduced the ice fraction, implying that hydrolyzable macromolecules disintegrated into non-ice-active fragments. Together with the heat resistance, this points to the presence of ice-active polysaccharides, either free or bound to the surface of microorganisms or debris. Adding calcium chloride (1.6 g L^–1^) to the 0.2 μm filtered *T. pullulans* aliquot did not change the ice fraction, but subsequent filtration led to a substantial reduction (Figure S2E). This suggests that INMs in the dissolved fraction may be acidic polysaccharides, as they tend to form microgels with a cross-linked structure in the presence of divalent Ca^2+^ cations, resulting in particle sizes greater than 0.2 μm.^84,85^ We hypothesize that particulate INMs, though not explicitly investigated here, may readily exist as such three-dimensional networks. Similarly, a standard solution of alginic acid, a commercially available acidic marine polysaccharide with the highest INA among those tested (Figure S2F), showed comparable behavior to the microorganism culture aliquots. As other cultures did not provide enough material only *T. pullulans* was characterized in more detail. Given the consistency of results across the different microbial cultures regarding physical treatments (Figure 2), we assume that these observations allow for a certain level of generalization.
(ii) To investigate polysaccharidic INA, standard polysaccharides commonly found in both, the marine and terrestrial environment, including laminarin, λ-carrageenan, κ-carrageenan, alginic acid (long- and short-chained), agar, cellulose, xanthan gum, and a lipopolysaccharide from *P. aeruginosa* were examined in a manner similar to the microbial samples. INA was found for all selected polysaccharide solutions. However, the different polysaccharides revealed a broad variability in terms of INA (Figure S3). The most ice-active polysaccharides with regard to observed onset temperature and number of INMs per dry mass were long-chained alginic acid and agar. It is important to note that, in the case of alginic acid, the qualitative experiments conducted (same as described under (i)) align closely with the observed behavior of the microorganism culture aliquots. Furthermore, agar and alginic acid are both negatively charged polysaccharides with gelling properties. However, other negatively charged polysaccharides, such as λ-carrageenan and κ-carrageenan, and neutral polysaccharides (cellulose, laminarin, lipopolysaccharide) showed an up to several orders of magnitude lower number concentration of INMs per dry mass. Additionally, an influence of the molecular weight is indicated as long-chained alginic acid (molecular weight: 100–200 kDa, degree of polymerization: 500–1000) was more ice-active than short-chained alginic acid (molecular weight: 2–40 kDa, degree of polymerization: 60–200). In summary, the sequence of monosaccharides within the polysaccharidic structure and their molecular weight had an influence on the INA of polysaccharides, although these features are obviously not the only relevant factor determining the INA. The effect of the secondary and tertiary three-dimensional structure on the catalysis of the ice nucleation has not been clarified. Previous studies also found an influence of chain length^86^ and particle size^87^ of biopolymers on their INA. However, the exact structures or features causing ice nucleation activity of biopolymers in general and in particular for polysaccharides remains elusive and more detailed experiments are required in the future.
(iii) Polysaccharides in the cultivated microbial samples were detected in the dissolved and particulate phases, here referred to as total combined carbohydrates (TCCHO) (Table S3). The monosaccharide composition (Figure S4) revealed a complex mix of carbohydrates in all microorganisms contributing 4–66% to the total organic carbon. In the cultures of *T. pullulans*, *T. striatum*, and *N. diffluens*, the highest fraction of TCCHO was rather found in the particulate phase (76–91%) than in the dissolved phase (9–24%), which fits well to the strong reduction of INA after the filtration of these samples. Based on the monosaccharide footprint, strong indications for the presence of agar or agar-like ice-active polysaccharides within the microorganisms were found, while the presence of alginic acid could be excluded by the lack of mannuronic acid, a monosaccharide unit released during the acidic hydrolysis of alginic acid. The normalization of the INM number site density (eq S1) of the different microorganisms on a specific chemical measure—the carbohydrate carbon mass C-TCCHO—revealed a significant decrease of its spread between the different microbial samples from three to one order of magnitude (Figure 3, Figure 2A), which strongly indicates a causal relationship between C-TCCHO and INA. Additionally, a very high agreement of the INA of eukaryotic microorganisms and those of the marine polysaccharides alginic acid and agar could be found from the almost identical INM number site density normalized to C-TCCHO (Figure 3). As a result, we used INM number density normalized to C-TCCHO to parametrize INP resulting from polysaccharidic INMs in the marine organic aerosol (parametrization hereafter called HSZ25).

In summary, the chemical identity of the INMs produced by the investigated marine eukaryotic microorganisms consists of polysaccharides either in their free form or bound to the microbial cell or fragments thereof. As described above, three arguments substantiate this conclusion: (i) targeted qualitative physical and chemical experiments elucidate a polysaccharidic nature of the INMs in the analyzed microbial samples, (ii) standard polysaccharides, which are also found in the marine and terrestrial environment, showed INA and behaved similarly compared to microbial samples, and (iii) TCCHO could be quantified (see the Experimental Methods section) and linked to observed INA. The spread of INM concentration of the various microbial samples reduces to less than 2 orders of magnitude (compare Figures 2A and 3). The similarity between the INA of microbial and standard polysaccharide samples and reduced spread, when normalized to C-TCCHO, strongly suggests polysaccharidic INMs as the cause of the observed freezing behavior in the microbial samples. Furthermore, with the conducted experiments we can rule out other potential macromolecular candidates, such as proteins, for inducing the observed ice formation (Figure 2). As a further conclusion, since marine INA polysaccharides (including alginic acid and agar) are also produced by other marine organisms, including globally distributed macro- and microalgae,^88−91^ their importance is much greater on a global scale. Since several INA polysaccharides analyzed in this study show similar freezing behavior when normalized to C-TCCHO (Figure 3) and have a variety of biological sources, it is justified to consider the INA polysaccharides directly rather than the individual microorganisms to assess their global significance. The investigation of these biological sources, their emissions, vertical transport, and oceanic and atmospheric concentration of INA polysaccharides in detail is an interesting subject of future research. Finally, since we found different ice-active polysaccharides, which entails a certain diversity that is also reflected in the spread of *n*_m, C-TCCHO_ in Figure 3, the individual curves of *n*_m, C-TCCHO_ for each eukaryotic microorganism and marine polysaccharides are parametrized with the CNT-based model (the method is described in Section S6 SI, and the results are given in Table S4). In the following, we use the derived parametrization of *T. striatum* and declare it as HSZ25.

### 3.2 Marine Polysaccharides Are Relevant Contributors to the INP Population in Remote Marine Regions Worldwide

**Figure 4:**
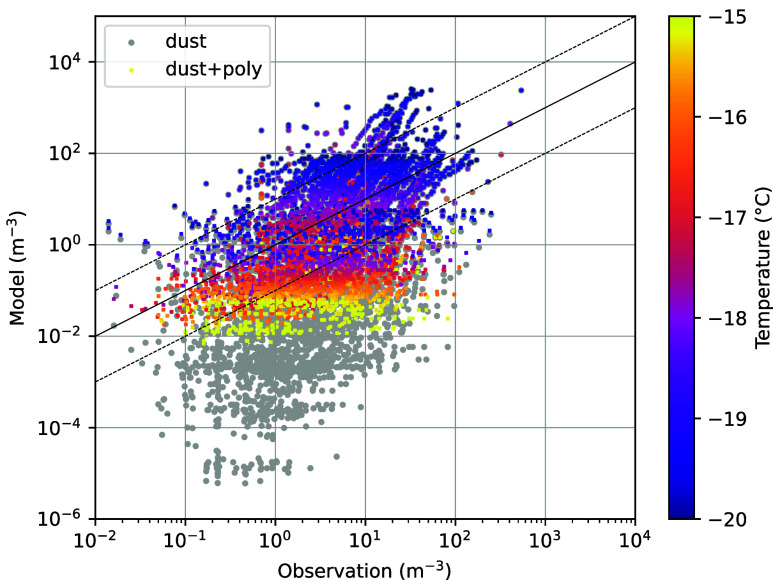
Modeled against observed INP concentration between −15 and −20 °C. Measurement data from 14 different campaigns (*n* = 5364; see Tab S5 and Figure S6 for references and regional coverage) in predominantly marine air masses was used. These were compared to annual mean INP concentrations derived from modeled mineral dust and sea salt concentrations simulated with a global model. For mineral dust INPs, the parametrization by Niedermeier et al.,^70^ and for marine polysaccharide INPs, the parametrization derived in this work was applied. Gray dots show the comparison between modeled mineral dust INPs and observation, whereas colored dots (color-code by observation temperature) present the sum of modeled mineral dust + marine polysaccharide INPs. For orientation, the figure shows the 1:1 line (solid) and the 1:10 and 10:1 lines (dashed).

The importance of marine polysaccharidic INMs is estimated by applying the HSZ25 parametrization to marine polysaccharide concentrations derived from simulated sea salt mass and measured polysaccharide fractions in sea spray aerosol (0.5% in accumulation mode and 0.1% in coarse mode, see Sect. 2.3), in addition to INPs contributed by mineral dust.^70^ The resulting INP concentrations were compared to worldwide available observations in the marine atmosphere (−20 to −15 °C: Figure 4 and Table S5, all observed temperatures: Figure S5). Note that by using only mineral dust and marine polysaccharides as INP types, the model is expected to underestimate the observed INP concentrations for temperatures >−15 °C (Figure S5). For such high temperatures, other INP types (e.g., proteinaceous INMs^92^) may account for the majority of INPs, which cannot be determined from the present study. Furthermore, terrestrial INPs other than mineral dust (e.g., terrestrial biological INPs) might contribute, as well.

Remarkably, despite its low concentration in remote marine regions mineral dust still provides a noticeable ubiquitous contribution to INPs at *T* < −15 °C even on the Southern Hemisphere consistent with previous model studies.^4,55,56^ Considering the INMs from marine polysaccharides in addition to mineral dust INPs, the modeled total INP concentrations increased substantially for *T* > −20 °C, leading to a better agreement with the observations (Figure 4, Table S5). In the temperature range of −15 to −20 °C, the fraction of modeled INP concentrations that are within a factor of 2 and a factor of 10 to the observations, increases from 10 and 37% (when only accounting for mineral dust INPs), to 15 and 60%, respectively, for all Southern Ocean campaigns (Table S5). For individual campaigns, particularly in the remote Southern Ocean, even better agreement was found. Due to the higher abundance of mineral dust in the Northern Hemisphere, the relative contribution of nondust INPs is lower and the average simulation improvement is weaker for all considered INP data sets (agreement within a factor of 10 increasing from 52 to 63%). Variation of the polysaccharide content in the modeled sea spray aerosol in both modes to 0.05 and 0.5%, respectively, as lower and upper estimates, leads to an agreement within a factor of 10 of 47 and 86% for the Southern Ocean campaigns and of 56 and 77% for all campaigns (Table S5).

The concentration of marine INPs in sea spray aerosol can be represented by a previously developed parametrization that uses sea spray aerosol surface area as proxy^59^ (hereafter M18). However, M18 was developed based on samples that were not heat-labile, indicating only minor contributions from, e.g., proteinaceous compounds. By applying the M18 parametrization to the modeled sea salt concentration instead of HSZ25, a similar improvement (i.e., INPs in addition to mineral dust INPs) can be obtained, primarily with better agreement to observations (fraction in factor of 2 and factor of 10, see Table S5). To compare the performance of the two parametrizations, the increase in agreement within a factor of 10 (in addition to mineral dust only) is used (eq 1). Across the different Southern Ocean data sets and in the temperature range of −15 to −20 °C, the HSZ25 parametrization reproduces between ∼30 and 115% of this increased agreement within a factor of 10 found for the M18 parametrization (44% on average for all observational data sets including Northern Hemisphere). The reproduced fraction is still 10–100% in the Southern Ocean (19% for all campaigns) for a polysaccharide content of 0.05%, increasing toward the upper bounds of polysaccharide content of 0.5 to 100–120% in the Southern Ocean (104% for all campaigns) as shown in Table S5. Hence, polysaccharides, which are the only considered compound group in HSZ25, can explain a large fraction up to all marine INPs (as seen in M18) within −15 to −20 °C. The discrepancy between the two parametrizations might be caused by their general uncertainties (e.g., total marine INPs in SSA can include other compounds than the polysaccharides applied in this study), the assumed polysaccharide content and its spatiotemporal variability that is currently not covered by the applied model, and other INP types that might have been present in the samples that M18 is based on (e.g., more efficient INA polysaccharides).

**Figure 5:**
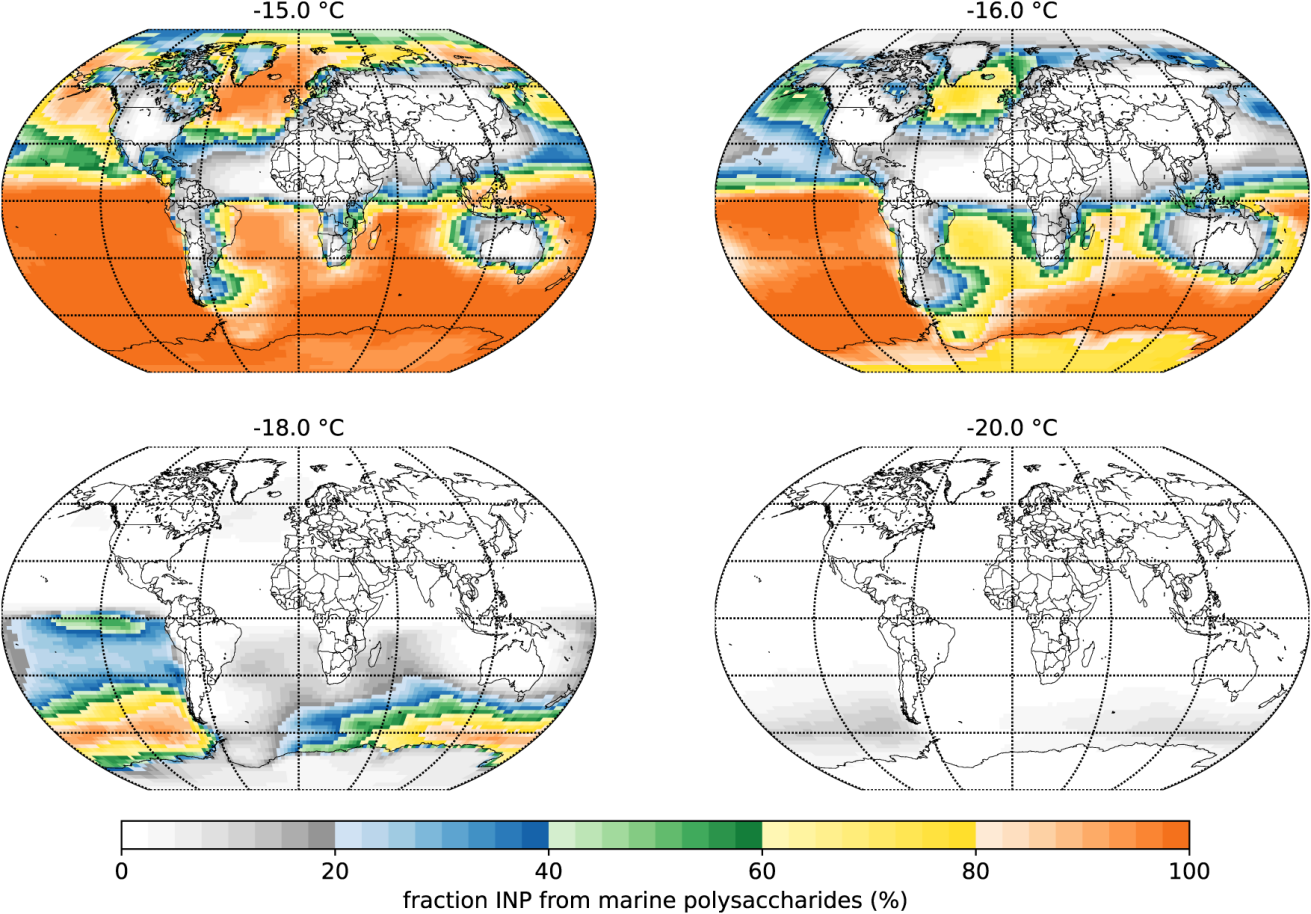
Percentage of modeled polysaccharide-based marine INPs in the sum of modeled INPs (mineral dust + marine polysaccharides) in the lowermost model layer for different temperatures. Annual mean INP concentrations were derived from mineral dust concentrations and from polysaccharides estimated based on sea salt concentrations simulated by a global model.

At the higher end of the relevant temperature range (−15 to −16 °C), on annual average, the INP concentrations resulting from marine polysaccharides are comparable to or exceed the INP concentrations from mineral dust in most parts of the marine atmosphere and in particular on the Southern Hemisphere (Figure 5). For a lower temperature, the importance of the INPs from marine polysaccharides decreases as mineral dust increases. Over the remote oceans of the Southern Hemisphere, the modeled INPs from marine polysaccharides are comparable to INPs from mineral dust down to about −19 °C and thus represent an important contributor to INPs in remote marine regions in a temperature range relevant for mixed-phase clouds. For lower temperatures, mineral dust INPs always dominate.

### 3.3 Implications for Modeling of INPs

In the present study, we show that in the temperature range from −15 to −20 °C, a significant part of the temperature-dependent INP number concentration in the marine atmosphere can be described based on a single relevant chemical component, ice-active marine polysaccharides. The CNT-based physical foundation of the chosen parametrization function has advantages for the application in atmospheric models as it reproduces observed INP-type-specific natural limits, i.e., realistic representation of freezing onset and implicitly comprising an upper limit of resulting INPs. Hence, possibly invalid extrapolation of log-linear parametrizations into temperature ranges that are not supported by measured data is avoided. Further, the concept is applicable to other INP sources and types, e.g., land-based biological INMs or specific ice-active mineral dust components, and allows for a time-dependent description of freezing using the nucleation rate (applying derived parameters of the contact angle distribution) instead of ice nucleation site density.

As a perspective, the present study pilots a way toward enhanced process understanding of ice formation by describing the total INP concentration using a combination of ice-active chemical classes that are causally linked to ice formation instead of using proxy aerosol properties that are only correlated with INA. Although this concept requires research on ice-active chemical classes, their emission pathways, and their atmospheric distribution, it would enable atmospheric models to directly relate ice formation and cloud glaciation to the occurrence of the relevant causes with realistic spatiotemporal variability of INPs. Modeling approaches for emission of, e.g., mineralogy of mineral dust^93^ and marine biological macromolecule classes do exist^94^ allowing for such a causal description of the total INP concentration.

The significant role of a natural biogenic aerosol constituent for the atmospheric INP budget highlights the importance of the coupling between biosphere and atmosphere in the Earth System. While anthropogenic aerosol emissions are expected to decrease with mitigation strategies to climate change,^95^ natural aerosol particles will become even more important for cloud microphysics. Clouds in a cleaner environment, i.e., with a low droplet number (“aerosol limited regime”), are more sensitive to variability in aerosol number concentration.^96,97^ Furthermore, it is important to note that mineral dust emissions were and are subject to regionally differing changes.^98−100^ Overall, as anthropogenic sources do not contribute significantly to the atmospheric INP budget,^101,102^ the number relation between cloud droplets and INPs might change in a less polluted atmosphere, with implications for cloud phase in mixed-phase clouds and therefore on cloud radiative forcing.

## Supporting Information Available

Additional details of the experimental setup, methods, and modeling approach (PDF)
