# Supplementary material for: Polysaccharides—Important Constituents of Ice-Nucleating Particles of Marine Origin
Source: Environ Sci Technol. 2025 Mar 7;59(10):5098–108. doi: 10.1021/acs.est.4c08014 (PMC11924218; doi:10.1021/acs.est.4c08014)
Supplement: Supplementary file 1 — es4c08014_si_001.pdf [file es4c08014_si_001.pdf]

Supporting Information for Publication Polysaccharides - Important Constituents of Ice Nucleating Particles of Marine Origin

Susan Hartmann,[*,†,‡] Roland Schrödner,[*,¶,‡] Brandon T. Hassett,[§] Markus Hartmann,[†] Manuela van Pinxteren,[‖] Khanneh Wadinga Fomba,[‖] Frank Stratmann,[†] Hartmut Herrmann,[‖] Mira Pöhlker,[†] and Sebastian Zeppenfeld[‖,‡]

†*Department of Atmospheric Microphysics (AMP), Leibniz Institute for Tropospheric Research (TROPOS), Leipzig, 04318, Germany.*

‡*These authors contributed equally to this work.*

¶*Department of Modeling Atmospheric Processes (MOD), Leibniz Institute for Tropospheric Research (TROPOS), Leipzig, 04318, Germany.*

§*Department of Arctic and Marine Biology, UiT – The Arctic University of Norway, Tromsø, 9019, Norway.*

‖*Atmospheric Chemistry Department (ACD), Leibniz Institute for Tropospheric Research (TROPOS), Leipzig, 04318, Germany*

E-mail: susan.hartmann@tropos.de; roland.schroedner@tropos.de

Summary: 26 pages, 7 figures, 5 tables.

S1 Further explanations to Figure 1

Ice nucleation data is typically presented as nucleation site density per volume (n_v), surface area (n_s), or mass (n_m). In order to visualize the data using the same metric, here we chose n_m, it necessary to convert reported n_s and n_v into n_m. For the conversion from n_v to n_m, the total mass of material was required. Following Xi et al. (2021),[1] we estimated the total mass by employing the number of cells reported in the respective publication, the cell volume as given in the literature, and a assuming a cell density of $1\,\mathrm{gcm}^{-3}$. If the literature directly reported a cell volume, that volume was used. If the diameter along two perpendicular axes was reported, the shape of the cell was assumed to be a prolate spheroid and the volume calculated accordingly, and if only one cell diameter was reported, the cell shape was assumed to be a sphere. For the microorganisms reported in Creamean et al. (2020)[2] the respective values were taken from Kocur and Hodgkiss (1973),[3] Elshaded et al. (2004),[4] Sublimi et al. (2011),[5] and Tindall et al. (1984).[6] For the species in Ickes et al. (2020)[7] the values are taken from Olenina et al. (2006).[8] For the species in this study, the TOC mass was as the closest available parameter to the total material mass. For the conversion from n_s to n_m of microbial INMs, a cell density of $1\,\mathrm{g\,cm}^{-3}$ was assumed as before. Information on the shape and diameter of the cells was again taken from the literature, and the surface area of the cells and thus their volume was calculated accordingly. For the species reported by Haga et al. (2013, 2014),[9,10] Morris et al. (2013),[11] Jayweera and Flanagan (1982),[12] and Iannone et al. (2011)[13] the n_s data was extracted from Haga et al. (2014)[9] and converted with the geometric parameters therein. For the conversion from n_s to n_m of the mineral dusts we assume spherical particles with a diameter of 662 nm and certain densities. This diameter is the modal value of the particle volume size distribution (PVSD) of heavy dust episodes in the 10-year long dataset of aerosol particle measurements at Cabo Verde by Gong et al. (2022).[14] Since this represents the particle diameter that contributes most to the particle mass in natural mineral dust, it is a reasonable simplification in lack of PVSDs for the mineral dusts used in the cited studies. As the bulk density of mineral dust we used

2.6 g cm^{-3}[15] and for the specific minerals we used the average value reported in Anthony et al. (2001).[16] Since plagioclase is not a single mineral, the average of the densities of the endmembers of the plagioclase series (albite and anorthite) was used. As described above, the derivation of n_m follows a few assumptions and underlies uncertainties. The highest uncertainty follows from the ice nucleation experiments with maximum uncertainty of one order of magnitude. A variation of parameters assumed for the calculation of n_m such as particle diameter, density or cell count, is estimated to lead in maximum to a change of a factor of 2.5 and is therefore well below the uncertainties of the ice nucleation measurements.

S2 Microorganisms in the marine environment

To assess if actual Arctic marine air and surface water samples contained fungi and thraustochytrids, we deployed a Burkard cyclone spore trap (Rickmansworth, UK) for at least two hours (16.51 Lmin^{-1}) from the front of the forecastle deck (∼8 m above seawater) of the R/V Helmer Hanssen in November 2017. To eliminate ship-borne contamination, air was only sampled when there was a prevailing headwind. Microcentrifuge tubes containing sampled air particulate was centrifuged at 10,000 RPMs for 5 minutes, followed by a DNA extraction with the DNeasy PowerMax Soil Extraction kit (Qiagen), according to manufacturer's instruction. DNA was subsequently amplified with the TAReuk454FWD1 5'-CCAGCASCYGCGGTAATTCC-3' and TAReukREV3 5'-ACTTTCGTTCTTGATYRA3' primer set[17] and sequenced on the Illumina MiSeq. Data was processed using Mothur v.1.43.0,[18] closely adhering to suggested processing parameters.[19]

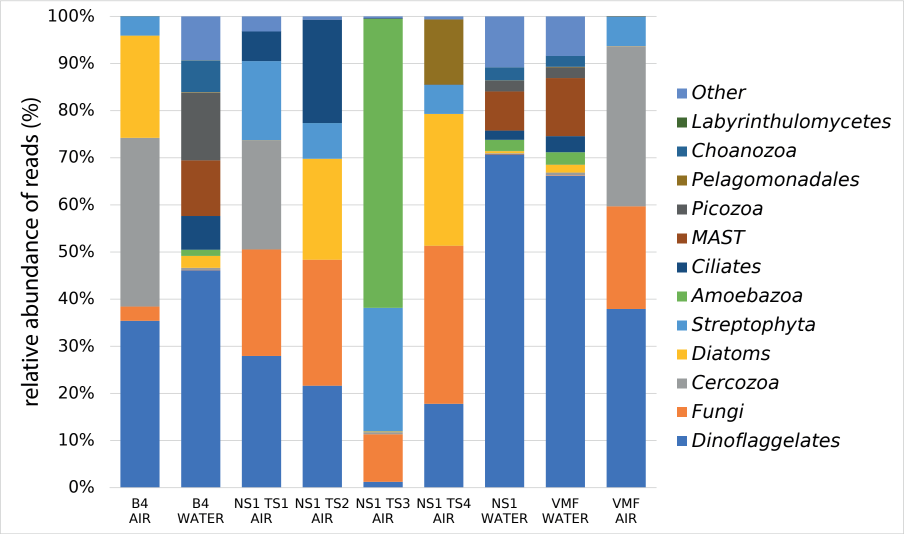

Figure S1: Relative sequence abundance of microbial eukaryotes in surface water and air taken at different sampling sites in November 2017 during the Polar Night (Van Mijenfjorden, VMF) onboard the R/V Helmer Hanssen, details described in Table S1. Fungi are the second most frequently detected in air samples. Thraustochytrids are classified under Labyrinthulomycetes, whose existence could be verified in samples: surface water: B4, NS1, VMF and air: NS1 TS1, NS1 TS3.

Table S1: Site information for polar night cruise 2017 on the R/V Helmer Hanssen, detailing site names, corresponding metadata, and the volume of water and air sampled.

**Site**	**Type**	**GPS coordinates**	**Date**	**Time**	**Volume sampled**
NS1 Transect 1	Air	N79 41.30 E1837.79	19. Nov 17	12:13-14:13	1,981 L
NS1 Transect 2	Air	N79 26.75 E1957.60	19. Nov 17	14:13-16:13	1,981 L
NS1 Transect 3	Air	N79 26.75 E1957.60	19. Nov 17	16:13-18:13	1,981 L
NS1 Transect 4	Air	N79 26.75 E1957.60	19. Nov 17	18:13-20:27	2,212 L
NS1	Surface Water	N80 25.62 E1525.03	19. Nov 17	NA	1 L
B4	Air	N77 26.73 E2734.43	22. Nov 17	16:24-21:30	5,052 L
B4	Surface Water	N77 25.65 E2734.32	22. Nov 17	NA	1L
Van Mijenfjorden	Air	N77 49.44 E1635.67	24. Nov 17	18:07 (>5 h)	>5,052 L
Van Mijenfjorden	Surface Water	N77 49.44 E1635.67	24. Nov 17	NA	1L

S3 Collection and cultivation of microorganisms

Cultures of heterotrophic eukaryotic microbes (*T. striatum*, *T. pullulans*, *N. diffluens*, *P. chrysogenum*) were established by sampling a variety of environments, including nearshore Arctic sediment in Tromsø and Arctic marine plankton. These samples were placed on PmTG (peptonized milk, tryptone glucose) agar media amended with seawater, penicillin G, and streptomycin sulfate antibiotics to inhibit bacterial growth. These cultures were incubated until microbial growth was observed. Subcultures were made, until an axenic culture was established. These cultures were maintained at 4 °C until DNA extraction. DNA was extracted from cultures using the DNeasy Powermax Soil extraction kit, per manufacturer instructions. For hyphal-forming fungi, the ITS locus was amplified using PCR with the ITS4-ITS5 primer set.[20] For unicellular thraustohytrids, the 18S rRNA gene was amplified using the NS1-NS4 primer set.[20] Amplicons were purified using the PureLink PCR Purification Kit (ThermoFisher) and subsequently commercially sequenced by GeneWiz (United Kingdom). Sequences from cultured isolates were deposited in NCBI's nucleotide database (Table S1, S2). Pure cultures were grown in liquid PmTG media for approximately two weeks in 1 L bottles. After two weeks, biomass was concentrated by centrifugation and washed several times in Dulbecco's Phosphate Buffered Saline (DPBS, Biowest) and subsequently resuspended for ice nucleation analysis. To serve as a control, liquid PmTG was included in the ice nucleation spectra analysis to account for any residual left after extensive washing.

Table S2: Sequence results and deposited accession number of heterotrophic eukaryotic microbes cultured.

**Isolate**	**Affinity**	**NCBI Accession**	**Locus**	**GPS coordinates**
B24	*Thraustochytrium striatum*	MT218310	18S	N69.663 E18.906
M2	*Tausonia pullulans*	MN967076	ITS	N69.631 E18.909
M3	*Naganishia diffluens*	MN967077	ITS	N69.631 E18.909
ZP1	*Penicillium chrysogenum*	MT218320	ITS	N78.984 E11.775
ZP2	*Penicillium chrysogenum*	MT218321	ITS	N78.984 E11.775

S4 Ice nucleation activity of pure and treated microbial samples

The ice nucleation activity was analyzed with the droplet freezing assay INDA (Ice Nucleation Droplet Array).[21,22] The liquid microbial samples made from isolated and cultivated fungi and protist are placed into a 96-PCR plate (Brand GmbH & Co. KG, Wertheim, Germany) with a volume of 50 µL per tube. The PCR plate is sealed and placed in a thermostat that cools the sample at a rate of approx. $1\,\mathrm{K\,min^{-1}}$ with a temperature uncertainty of 0.5 K. The number of frozen aliquots per temperature interval of 0.1 K is recorded by a camera and the temperature dependent ice fraction, which is the number of frozen per total number of aliquots, is derived. To cover a wider temperature and concentration range, a dilution series using DPBS buffer is analyzed for all samples. The DPBS buffer exhibited no significant ice-nucleating properties (Figs. 2, S2). The INM number density can be calculated using[23]

$$\mathrm{n_x}(T) = \frac{-\ln(1-\mathrm{f_{ice}})}{\mathrm{x}}, \tag{S1}$$

whereas x can be related to sample volume (x=v), total particle surface area (x=s) or particle mass (x=m). The heat-sensitivity of the INMs was tested by heating the PCR plates of the different samples at 95 °C for one hour and repeating the INDA measurements. Further, for starting from the same stock suspension all microbial samples were filtered with pore diameter of 0.2 µm (PTFE, Whatman™) to classify the size of INMs and whether they are bound to the microbial cells.

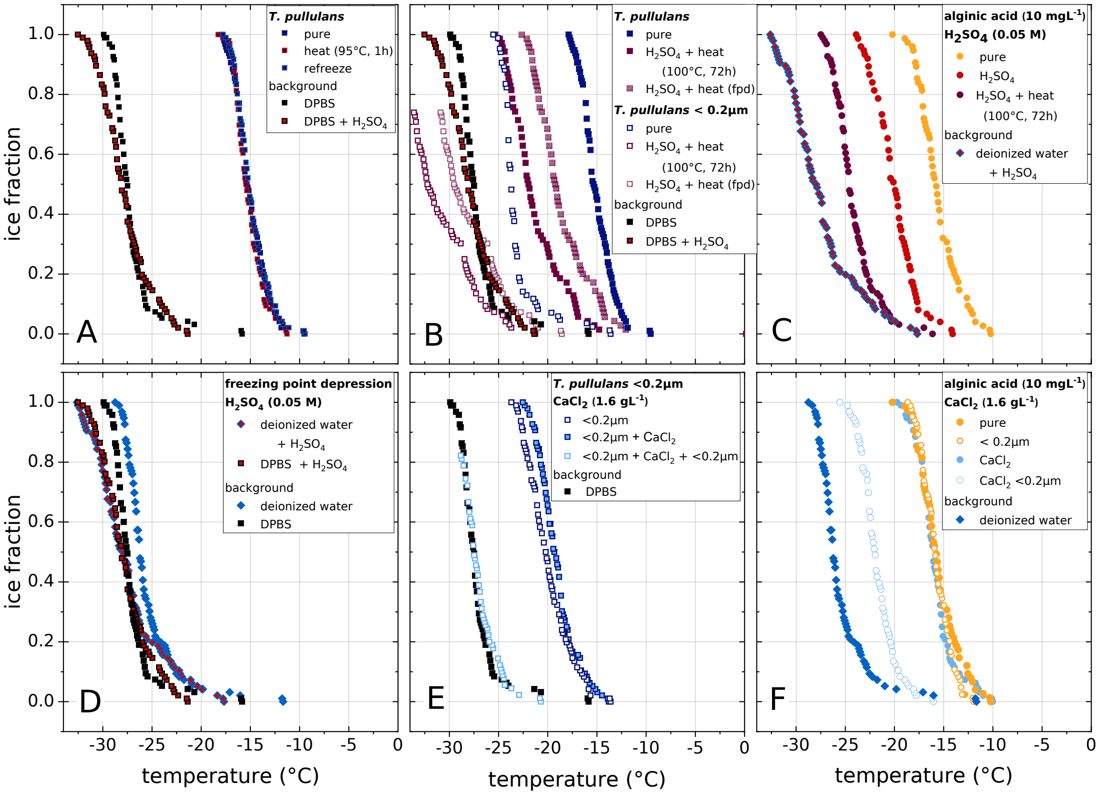

Figure S2: Newly developed and targeted investigation of chemical identity of INMs. As a representative microorganism, the chemical identity of the INMs in *T. pullulans* was analyzed. Tests with a solution of the ice-active standard polysaccharide alginic acid verified the applicability of the tests. A&D: standard methods (heat treatment and refreeze) and background measurements of the applied buffer DPBS (Dulbecco's Phosphate Buffered Saline, Gibco) and deionized water (resistivity $\geq$ 18.2 MΩcm), together with 0.05 M H_2SO_4 to study their effect on the freezing point depression, the decomposition of ice-active polysaccharides into non-ice active monosaccharides. B&C: Microgel formation after an addition of 1.6 gL^{-1} $CaCl_2$ with subsequent filtration (pore size <0.2 µm; E, F) lead to a significant reduction of the observed INA. The freezing point depression caused by added substances for chemical treatment were considered.

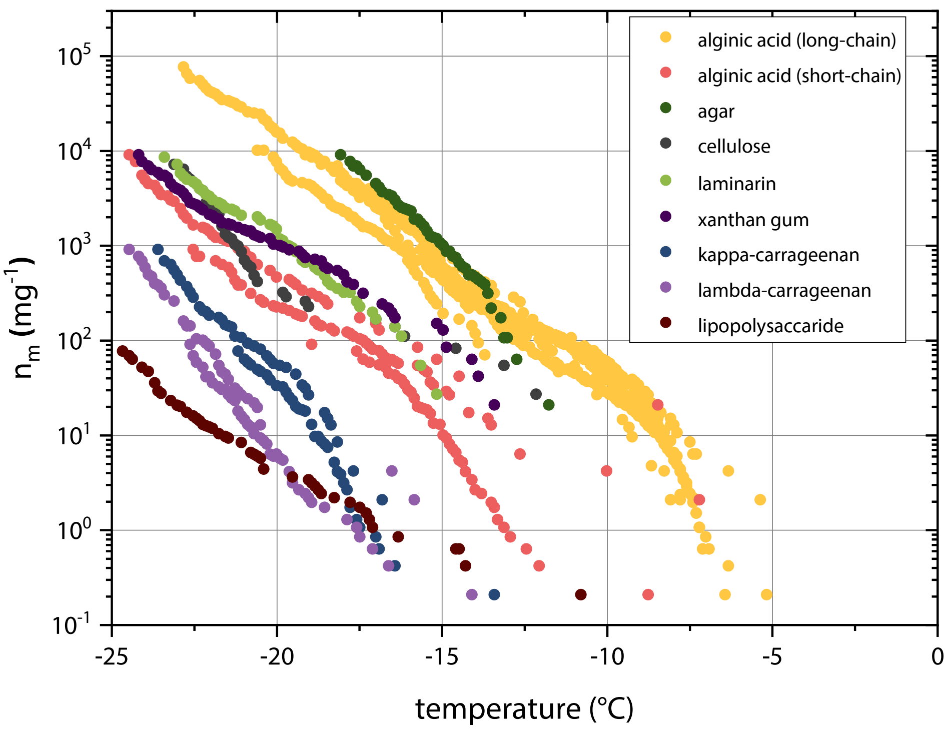

Figure S3: Ice nucleation active polysaccharides from terrestrial (cellulose, xanthan gum, lipopolysaccharide) and marine origin (alginic acid, agar, laminarin, kappa- and lambda-carageenan). Temperature dependent ice nucleation site density normalized to mass of polysaccharides n_m.

S5 Chemical analyses of microbial samples and polysaccharide standards

Dissolved (DCCHO, <0.2 µm) and particulate (PCCHO, >0.2 µm) combined carbohydrates - contained within the microorganism cultures, commercial polysaccharide standards and DPBS solution (blank) - were analyzed on a Dionex ICS-3000 high-performance anion exchange chromatography with pulsed amperometric detection (HPAEC-PAD) equipped with a Dionex CarboPac PA20 analytical column (3x150 mm) and a Dionex CarboPac PA20 guard column (3x30 mm) as described elsewhere.[24] HPAEC-PAD is a robust instrument to

detect monosaccharides and derivatives in very low (µg L^{-1} range) concentrations. Overlapping signals from other organic molecules, such as amino acids, hardly occur within the chromatograms unless they contain a high number of oxidizable functional groups (e.g., hydroxyl, aldehyde) within the same molecules and appear in much higher concentrations than the saccharidic compounds. DCCHO and PCCHO were calculated as the sum of the following identifiable monosaccharides and derivatives released after an acid hydrolysis (0.8 M HCl, 100 °C, 20 h): fucose (Fuc), rhamnose (Rha), arabinose (Ara), galactose (Gal), glucose (Glc), xylose (Xyl), mannose (Man), glucosamine (GlcN), galactosamine (GalN), muramic acid (MurAc), galacturonic acid (GalAc), glucuronic acid (GlcAc), mannuronic acid (ManAc). The acid hydrolysis step is required to break the chemical bonds within large polysaccharidic macromolecules resulting into individual monosaccharide units contained within, which can be subsequently detected and quantified with the exisiting analytical protocol using HPAEC-PAD.[24] Total combined carbohydrates (TCCHO) represents the sum of DCCHO and PCCHO.

Table S3: Carbon contained within dissolved (D), particulate (P), total combined carbohydrates (C-TCCHO) and total organic carbon (TOC) in the microorganism cultures ($mg\,C\,L^{-1}$). b.d.=below detection limit. [1]DPBS=Dulbecco's phosphate-buffered saline, used as blank; [2]TOC values were corrected by DPBS buffer. Ultrasonification is abbreviated to US.

Culture		Glc	Gal	Man	Xyl	Rha	Fuc	Ara	GalN	GlcN	GalAc	GlcAc	sum	C-TCCHO	share (%)	TOC[2]	C-TCCHO/ TOC (%)
P. chryso-genum	D	2.2	3.4	1.7	0.08	b.d.	0.3	0.03	0.03	2.3	b.d.	0.003	10.1	11	96	288	4
(culture ZP1, undiluted)	P	0.3	0.02	0.02	0.04	0.004	b.d.	0.001	0.001	0.01	b.d.	0.001	0.4		4		
P. chryso-genum	D	7.4	2.1	1.4	0.08	b.d.	b.d.	0.01	0.03	0.28	b.d.	b.d.	11.3	13	90	121	11
(culture ZP2, undiluted)	P	0.8	0.2	0.1	0.08	0.002	0.002	0.11	0.001	0.04	b.d.	b.d.	1.3		10		
T. pullulans	D	8.4	12.5	15.4	1.4	b.d.	0.71	0.05	0.04	2	b.d.	0.09	41	178	23	270	66
(undiluted)	P	73	23	23	4.7	b.d.	1.2	b.d.	0.09	11.4	0.49	1	138		77		
T. pullulans	D	5.6	4.3	7.2	0.84	b.d.	0.32	0.02	0.03	0.53	b.d.	0.05	19	96	20	388	25
(undiluted, US treatment)	P	45	15	7.7	1.9	b.d.	0.47	0.04	0.1	6.9	b.d.	0.29	77		80		
N. diffluens	D	15	3.5	8.3	1.7	b.d.	0.87	0.05	0.08	1.1	b.d.	0.33	31	204	15	1169	17
(undiluted)	P	75	54	25	7.8	b.d.	b.d.	b.d.	b.d.	6.6	0.38	4	173		85		
N. diffluens	D	3.5	0.59	1.3	0.35	b.d.	0.09	0.01	0.01	0.29	b.d.	0.13	6.3	54	12	284	19
(dilution 1:10)	P	32	6.9	4.7	1.5	b.d.	0.07	b.d.	b.d.	1.9	0.1	0.66	48		88		
N. diffluens	D	0.3	0.07	0.05	0.03	b.d.	b.d.	b.d.	b.d.	0.03	b.d.	0.01	0.5	5.6	9	40	14
(dilution 1:100)	P	2.9	1.9	0.16	0.13	b.d.	b.d.	b.d.	b.d.	0.08	b.d.	0.02	5.1		91		
T. striatum	D	5.7	3.8	1.5	0.78	0.05	0.13	0.05	0.08	0.4	b.d.	0.11	13	53	24	204	26
(undiluted)	P	7.8	28.9	1.2	0.92	0.1	0.16	0.07	0.09	0.71	b.d.	0.18	40		76		
T. striatum	D	2	0.6	0.4	0.32	b.d.	0.05	b.d.	0.02	0.13	b.d.	0.18	3.7	17	22	78	22
(dilution 1:10)	P	6.8	3.4	0.91	1.2	0.24	0.14	0.03	0.03	0.33	b.d.	0.09	13		78		
**DPBS[1]**	D	0.009	0.001	b.d.	b.d.	b.d.	b.d.	b.d.	b.d.	0.001	b.d.	b.d.	0.01	0.01	100	-	-
	P	b.d.	b.d.	b.d.	b.d.	b.d.	b.d.	b.d.	b.d.	b.d.	b.d.	b.d.	0		0		

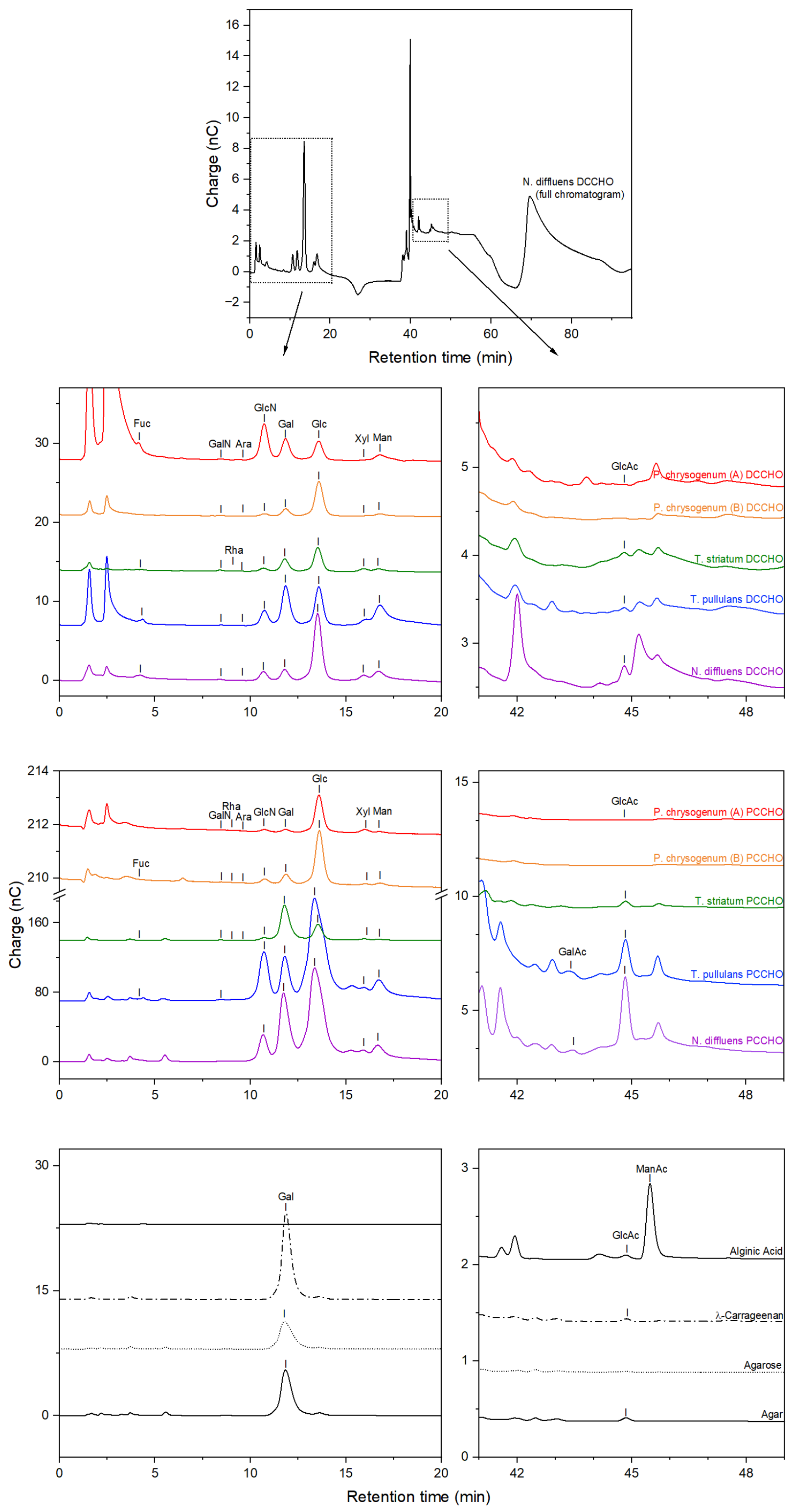

Figure S4: A complete example of an HPAEC-PAD chromatogram (A). Details of relevant parts of the chromatogram of the hydrolyzed DCCHO (B) and PCCHO (C) of *P. chrysogenum*, *T. striatum*, *T. pullulans*, *N. diffluens* and selected commercially available polysaccharide standards (D).

S6 INP parametrization based on classical nucleation theory

To describe the temperature-dependent INP number density, we apply parameterizations based on classical nucleation theory (CNT) for both, ice active marine polysaccharides derived in this study and mineral dust according to Niedermeier et al. (2015) (N15),[25] respectively. CNT describes the formation of critical ice clusters, which are preferentially formed at specific surface sites with the inherent property to catalyze the ice nucleation by reducing the critical energy barrier of phase transition from supercooled liquid water to ice. In this study, we use a CNT modeling framework which accounts for the Poisson distributed number and heterogeneity ice nucleation active surface features via the Gaussian contact angle distribution $p(\theta) = \frac{1}{\sqrt{2\pi\sigma_\theta}} \exp\left(-\frac{(\theta-\mu_\theta)^2}{2\sigma_\theta^2}\right)$ with the main parameters $(\mu_\theta, \sigma_\theta)$.[25–29] The INP number density can be described as

$$\mathrm{n_x}(T) = \mathrm{n_x^\star}(1 - \mathrm{P_{unf}}(\mathrm{T}, \mu_\theta, \sigma_\theta, \mathrm{dT/dt})) \tag{S2}$$

and

$$\mathrm{P_{unf}}(\mathrm{T}, \mu_\theta, \sigma_\theta, \mathrm{dT/dt}) = \int_0^\pi p(\theta) \exp\left(-\frac{\mathrm{s_{site}}}{dT/dt} \int_T^{T_0} \mathrm{j_{het}}(T, \mu_\theta, \sigma_\theta) dT\right) d\theta + C \tag{S3}$$

with specific average number of ice nucleation active surface features $\mathrm{n_x^\star}$ which can be related to mass (x=m) or surface area (x=s), temperature T, cooling rate $\frac{dT}{dt}$, surface area of specific ice nucleating surface site $\mathrm{s_{site}}$ and heterogeneous ice nucleation rate coefficient $\mathrm{j_{het}}(T, \theta_i) = \frac{kT}{h} n \exp\left(-\frac{\Delta F(T) + \Delta G(T) f(\theta_i)}{kT}\right)$. Here k and h are the Boltzmann and Planck constants, n is the number density of water molecules at the ice nucleation surface site. $\Delta F(T)$ represents the diffusion energy across the water-ice interface and $\Delta G(T)$ is the Gibbs free energy change for critical ice cluster formation. The spherical cap geometry of the ice cluster is described by $f(\theta_i) = \frac{1}{4}(2 + \cos\theta_i)(1 - \cos\theta_i)^2$. Since mineral dust is predominantly

insoluble, the surface-based INP number density approach and the contact angle distribution derived in Niedermeier et al. (2015)[25] (Table S4) are used. Considering the soluble and/or suspendable INMs produced by eukaryotic microorganism and standard polysaccharides we follow the mass-based approach.

Table S4: Parameters of CNT-based model to describe the ice nucleating spectra of analyzed eukaryotic microorganisms, the most efficient marine polysaccharides (Fig. 3) and mineral dust. The quality of the fit is indicated by the root mean square errors (rmse) of the logarithmic INP number density. The corresponding data sets of the CNT-based model can be found https://doi.org/10.5281/zenodo.10421589 and https://doi.org/10.5281/zenodo.10423597.

**Ice nucleation site density per mass of C-TCCHO $\mathbf{n}_{\mathrm{m,C-TCCHO}}$**				
**Microorganism**	$\mathbf{n}^{\star}_{\mathrm{m}}$ **(mg^{-1})**	μ_θ (°)	σ_θ (°)	**rmse**
T. striatum	7.20×10^4	50.4	4.4	0.3167
T. pullulans	1.46×10^5	63.8	6.2	0.2754
N. diffluens	1.11×10^6	63.6	6.4	0.8423
P. chrysogenum (ZP1)	1.77×10^9	99.8	11	0.1256
P. chrysogenum (ZP2)	5.42×10^3	53	3.8	0.3362
**IN-active polysaccharide**				
alginic acid	2.00×10^8	96	11.8	0.3800
agar	4.00×10^4	50	3.7	0.1242
**Ice nucleation site density per surface area of mineral dust $\mathbf{n}_{\mathrm{s}}$ (k-feldspar content 1-25%, representative for natural mineral dust, N15)[25]**				
**mineral dust**	$\mathbf{n}^{\star}_{\mathrm{s}}$ **(m^{-2})**	μ_θ (°)	σ_θ (°)	
	8.40×10^{10}	73.9	5.7	

For all derivations of the contact angle distributions, a cooling rate of $1\,\mathrm{K\,min^{-1}}$ and a specific surface area $s_{\mathrm{site}} = 1.28 \times 10^{-16}\,\mathrm{m}^2$ is assumed and for j_{het} parameterizations given in Zobrist et al. (2007)[30] are applied. We followed a two-step fit procedure: by minimizing the root mean square error of the logarithmic INP number density first the parameter of specific number of sites and the contact angle distribution $(\mu_\theta, \sigma_\theta)$ were fitted to the whole data set (Fig. 2), second the derived contact angle distributions are applied to determine $n^{\star}_{x}$ of the INM number normalized to C-TCCHO (Fig. 3, Table S4).

S7 Global model

The aerosol particles and their microphysical interaction is described using the aerosol microphysical module M7.[31] It considers 7 aerosol modes in 4 size sections (nucleation: $r < 5\,\text{nm}$, Aitken: $5\,\text{nm} < r < 50\,\text{nm}$, accumulation: $50\,\text{nm} < r < 500\,\text{nm}$, coarse: $r > 500\,\text{nm}$), of which the largest three modes are separated between soluble and insoluble aerosol particles. The modes are defined by variable median size of the aerosol particles and the fixed width of the modes. In each mode the number and the chemical composition of the aerosol particles is treated prognostically. The chemical composition can vary between the different modes. Considered aerosol species comprise mineral dust, sea salt, sulfate, black carbon, primary organic aerosol, and secondary organic aerosol. The model uses state-of-the-art schemes for the emission of mineral dust and sea salt.[32,33] In a comparison against ERA-Interim, over the Southern Ocean the modeled mineral dust is slightly underestimated, and sea salt and the aerosol optical depth (AOD) is overestimated.[33] However, against MODIS the modeled AOD in the northern and southern high latitudes fits well,[34] whereas ERA-Interim strongly underestimates the AOD.[33] Overall, the modeled sea salt and mineral dust concentrations, emission and deposition rates are within the range of other global models.[33] Within AeroCom Phase III, a low normalized mean bias and high correlation against different AOD observation datasets compared to other participating models was found for TM5.[35] The surface concentrations of mineral dust were evaluated within the CRESCENDO Earth System Models for the years 2000-2014,[36] in which TM5, as atmospheric chemistry model of EC-Earth, showed the lowest bias amongst all models and a high correlation. Furthermore, the model could reproduce main features of the seasonal cycle of mineral dust concentrations at various marine stations worldwide. The modeled sea salt concentrations were found to be close to observations at Mace Head and at Amsterdam Island, however showing less interannual variability than observed in the Southern Ocean.[37] The observational INP data comprises more than a decade, for which simulation data was not available for this study. The modeled sea salt and mineral dust concentration do not show a pronounced seasonal cycle in the marine

regions that were taken into account in this study. The modeled sea salt concentration shows only moderate variability. In contrast, the modeled mineral dust concentration, in particular on the Southern Hemisphere, varies considerably at the different sites depending on the frequency of long-range transported mineral dust from the continental source regions reaching the sites. Hence, we decided to use the annual mean INP concentration in the comparison against INP observations, instead of shorter periods covering e.g., only the months of the different campaigns, to avoid strong contribution of outlier mineral dust concentrations in the model data.

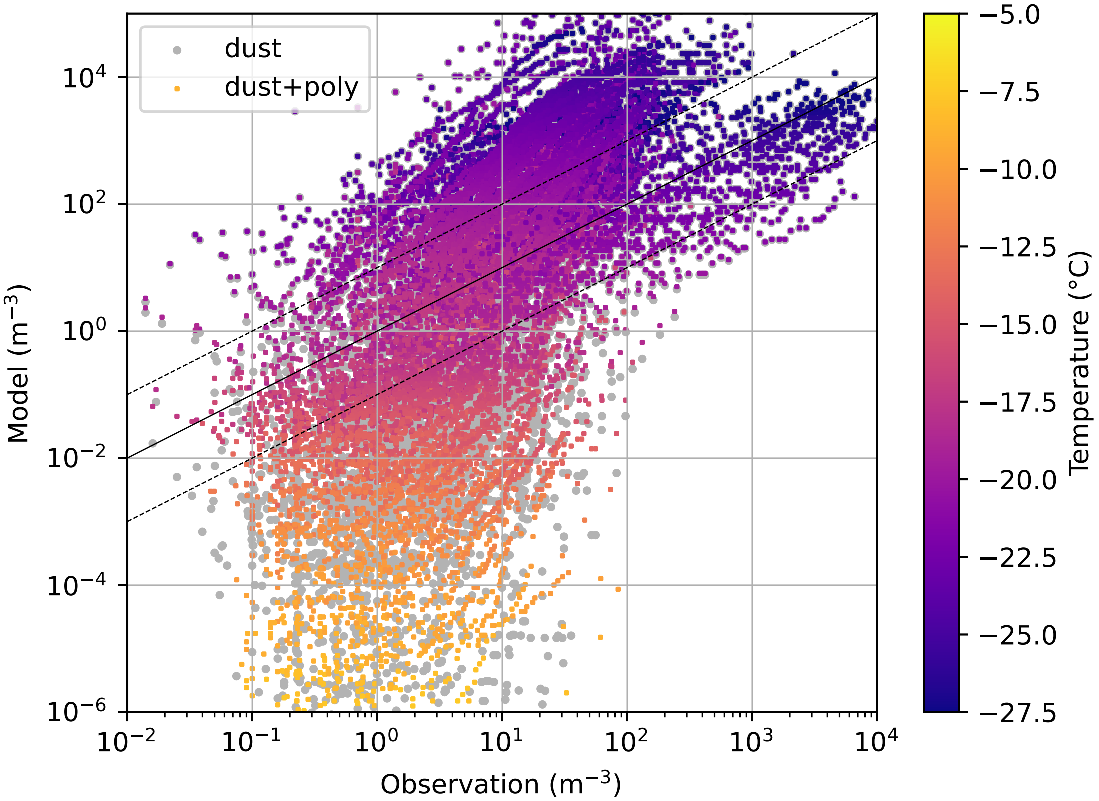

Figure S5: Modeled against observed INPs. INP concentrations in predominantly marine air masses measured worldwide during 14 different campaigns (see Table S5 and Fig. S6 for references and regional coverage). These were compared to annual mean INP concentrations derived from modeled mineral dust and sea salt concentration simulated with a global model. For mineral dust INPs, the N15 and for marine polysaccharide INPs, the HSZ25 parameterization derived in this work were applied, respectively. Grey dots show the comparison between modeled mineral dust INPs and observation whereas colored dots (color-code by observation temperature) present the sum of modeled INPs: mineral dust + marine polysaccharides. For orientation the figure shows the 1:1 line (solid) and the 1:10 and 10:1 lines (dashed).

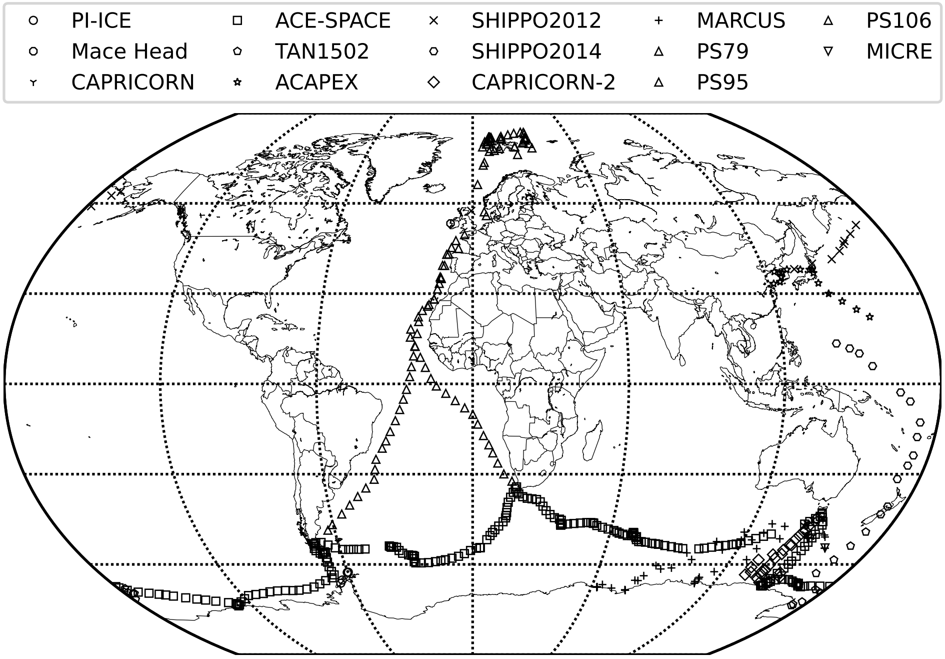

Figure S6: Location of measured INP data from different campaigns used in this study.

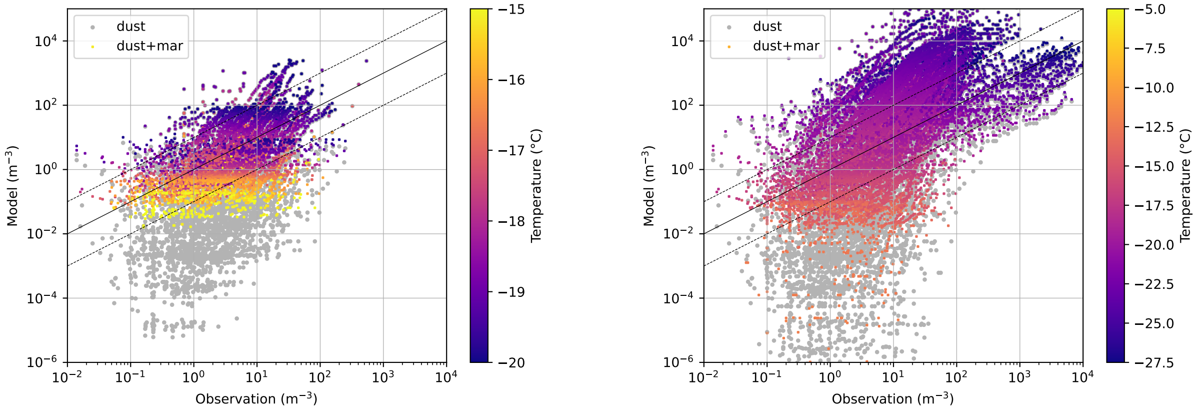

Figure S7: Same as Fig. 4 and S5 but using M18 as INP parameterization of marine INPs in addition to to mineral dust INPs.

Table S5: Modeled against observed INPs. Fraction of agreement of modelled and observed INP concentration within a factor of 2 and a factor of 10 for the available marine observational datasets, whose location are presented in Fig. S6. The numbers in brackets show the agreement within a factor of 2 and 10, respectively, for the lower (0.05 %) and upper (0.5 %) assumed polysaccharide content. The considered temperature range is -15 °C to -20 °C. References, season and location: PI-ICE[24] (austral summer, Antarctic Peninsula); CAPRICORN[38] (austral summer, Southern Ocean); ACE-SPACE[39] (austral summer, Southern Ocean); Mace Head[40] (boreal summer, land-based, N Atlantic Ocean), CAPRICORN-2[41] (austral summer, Southern Ocean), MARCUS[42] (austral summer, Southern Ocean), MICRE[43] (whole year, land-based, Macquarie Island), ACAPEX[44] (boreal winter, Yellow Sea, Sea of Japan, N Hemisphere, Western Pacific), PS79[45] (boreal spring, northern-southern hemisphere transect), PS95[46] (boreal fall, northern-southern hemisphere transect), PS106[47] (boreal summer, Arctic Ocean), SHIPPO2012 (boreal summer, NW Pacific), SHIPPO2014 (tropics, Coral Sea, trop. W Pacific), TAN1502 (austral summer, Southern Ocean) (datasets of the latter three campaigns: in courtesy of Paul J. DeMott).

**Campaign/ data set**	**# observations**	**Agreement model & observation (%)**						
		Mineral dust (N15) [25]		Mineral dust (N15) [25] + polysaccharides (HSZ25)		Mineral dust (N15) [25] + sea spray aerosol (M18) [40]		Marine INPs explained by poly-saccharides $F_{HSZ25,M18}$
		**f≤2**	**f≤10**	**f≤2**	**f≤10**	**f≤2**	**f≤10**	**f≤10**
**Southern Ocean**								
PI-ICE	153	21.6	76.5	33.3 (27-26)	87.6 (86-91)	26.8	88.2	95 (84-122)
CAPRICORN	59	22.0	66.1	32.2 (25-56)	89.8 (86-86)	52.5	86.4	117 (100-100)
ACE-SPACE	1061	8.6	32.7	11.5 (10-31)	53.1 (40-85)	30.7	83.3	41 (14-104)
CAPRICORN-2	38	18.4	65.4	18.4 (21-37)	92.1 (76-92)	36.8	92.1	100 (41-100)
TAN1502	43	23.3	53.5	32.6 (26-44)	76.7 (58-97)	44.2	97.7	53 (10-100)
MARCUS	388	18.3	46.6	27.8 (21-52)	85.1 (69-90)	51.5	88.9	91 (54-102)
MICRE	605	2.6	22.0	5.3 (4-15)	41.2 (29-83)	16.0	82.3	32 (11-101)
**All Southern Ocean**	**2347**	**10.3**	**36.9**	**15.0** (12-31)	**59.5** (47-86)	**31.0**	**84.8**	**47** (21-103)
**Other regions**								
PS79	844	24.6	61.8	25.1 (25-27)	62.9 (62-68)	27.0	67.3	20 (6-108)
PS95	153	16.3	59.5	16.3 (16-20)	62.1 (61-70)	18.3	68.6	29 (14-114)
PS106	1509	19.1	67.1	19.2 (19-21)	68.0 (67-71)	20.5	70.7	25 (6-114)
SHIPPO2014	30	13.3	50.0	13.3 (13-27)	56.7 (60-77)	26.7	76.7	25 (38-100)
SHIPPO2012	36	27.8	52.8	27.8 (28-31)	52.8 (53-56)	30.6	55.6	0 (0-100)
ACAPEX	70	12.9	34.3	12.9 (13-11)	34.3 (34-34)	11.4	34.3	-
RS Mace Head	375	21.3	58.4	21.6 (21-25)	65.3 (61-81)	24.8	78.4	35 (10-113)
**All campaigns**	**5364**	**16.1**	**51.6**	**18.3** (17-26)	**62.5** (56-77)	**26.3**	**76.3**	**44** (19-104)

References

(1) Xi, Y.; Mercier, A.; Kuang, C.; Yun, J.; Christy, A.; Melo, L.; Maldonado, M. T.; Raymond, J. A.; Bertram, A. K. Concentrations and properties of ice nucleating substances in exudates from Antarctic sea-ice diatoms. *Environ. Sci.: Processes Impacts* **2021**, DOI: 10.1039/D0EM00398K.

(2) Creamean, J. M.; Ceniceros, J. E.; Newman, L.; Pace, A. D.; Hill, T. C. J.; DeMott, P. J.; Rhodes, M. E. Evaluating the potential for Haloarchaea to serve as ice nucleating particles. *Biogeosciences* **2021**, *18*, 3751–3762, DOI: 10.5194/bg-18-3751-2021.

(3) Kocur, M.; Hodgkiss, W. Taxonomic Status of the Genus Halococcus Schoop. *Int. J. Syst. Evol. Microbiol.* **1973**, *23*, 151–156, DOI: https://doi.org/10.1099/00207713-23-2-151.

(4) Elshahed, M. S.; Savage, K. N.; Oren, A.; Gutierrez, M. C.; Ventosa, A.; Krumholz, L. R. Haloferax sulfurifontis sp. nov., a halophilic archaeon isolated from a sulfide- and sulfur-rich spring. *Int. J. Syst. Evol. Microbiol.* **2004**, *54*, 2275–2279, DOI: https://doi.org/10.1099/ijs.0.63211-0.

(5) Sublimi Saponetti, M.; Bobba, F.; Salerno, G.; Scarfato, A.; Corcelli, A.; Cucolo, A. Morphological and Structural Aspects of the Extremely Halophilic Archaeon Haloquadratum walsbyi. *PLOS ONE* **2011**, *6*, 1–9, DOI: 10.1371/journal.pone.0018653.

(6) Tindall, B.; Ross, H.; Grant, W. Natronobacterium gen. nov. and Natronococcus gen. nov., Two New Genera of Haloalkaliphilic Archaebacteria. *Syst. Appl. Microbiol.* **1984**, *5*, 41–57, DOI: https://doi.org/10.1016/S0723-2020(84)80050-8.

(7) Ickes, L.; Porter, G. C. E.; Wagner, R.; Adams, M. P.; Bierbauer, S.; Bertram, A. K.; Bilde, M.; Christiansen, S.; Ekman, A. M. L.; Gorokhova, E.; Hohler, K.; Kiselev, A. A.; Leck, C.; Mohler, O.; Murray, B. J.; Schiebel, T.; Ullrich, R.; Salter, M. E. The ice-

nucleating activity of Arctic sea surface microlayer samples and marine algal cultures. *Atmos. Chem. Phys.* **2020**, *20*, 11089–11117, DOI: 10.5194/acp-20-11089-2020.

(8) Olenina, I.; Hajdu, S.; Edler, L.; Andersson, A.; Wasmund, N.; Busch, S.; Göbel, J.; Gromisz, S.; Huseby, S.; Huttunen, M.; Jaanus, A.; Kokkonen, P.; Ledaine, I.; Niemkiewicz, E. Biovolumes and size-classes of phytoplankton in the Baltic Sea. *HELCOM Balt.Sea Environ. Proc.* **2006**, *106*, 144.

(9) Haga, D. I.; Burrows, S. M.; Iannone, R.; Wheeler, M. J.; Mason, R. H.; Chen, J.; Polishchuk, E. A.; Poschl, U.; Bertram, A. K. Ice nucleation by fungal spores from the classes Agaricomycetes, Ustilaginomycetes, and Eurotiomycetes, and the effect on the atmospheric transport of these spores. *Atmos. Chem. Phys.* **2014**, *14*, 8611–8630, DOI: 10.5194/acp-14-8611-2014.

(10) Haga, D. I.; Iannone, R.; Wheeler, M. J.; Mason, R.; Polishchuk, E. A.; Fetch, T.; van der Kamp, B. J.; McKendry, I. G.; Bertram, A. K. Ice nucleation properties of rust and bunt fungal spores and their transport to high altitudes, where they can cause heterogeneous freezing. *J. Geophys. Res. Atmos.* **2013**, *118*, 7260–7272, DOI: 10.1002/jgrd.50556.

(11) Morris, C. E.; Sands, D. C.; Glaux, C.; Samsatly, J.; Asaad, S.; Moukahel, A. R.; Goncalves, F. L. T.; Bigg, E. K. Urediospores of rust fungi are ice nucleation active at >-10 degrees C and harbor ice nucleation active bacteria. *Atmos. Chem. Phys.* **2013**, *13*, 4223–4233, DOI: 10.5194/acp-13-4223-2013.

(12) Jayaweera, K.; Flanagan, P. Investigations on biogenic ice nuclei in the arctic atmosphere. *Geophys. Res. Lett.* **1982**, *9*, 94–97, DOI: 10.1029/GL009i001p00094.

(13) Iannone, R.; Chernoff, D. I.; Pringle, A.; Martin, S. T.; Bertram, A. K. The ice nucleation ability of one of the most abundant types of fungal spores found in the atmosphere. *Atmos. Chem. Phys.* **2011**, *11*, 1191–1201, DOI: 10.5194/acp-11-1191-2011.

(14) Gong, X.; Wex, H.; Müller, T.; Henning, S.; Voigtländer, J.; Wiedensohler, A.; Stratmann, F. Understanding aerosol microphysical properties from 10 years of data collected at Cabo Verde based on an unsupervised machine learning classification. *Atmos. Chem. Phys.* **2022**, *22*, 5175–5194, DOI: 10.5194/acp-22-5175-2022.

(15) Möhler, O.; Field, P. R.; Connolly, P.; Benz, S.; Saathoff, H.; Schnaiter, M.; Wagner, R.; Cotton, R.; Krämer, M.; Mangold, A.; Heymsfield, A. J. Efficiency of the deposition mode ice nucleation on mineral dust particles. *Atmos. Chem. Phys.* **2006**, *6*, 3007–3021, DOI: 10.5194/acp-6-3007-2006.

(16) of America, M. S. Handbook of Mineralogy. https://handbookofmineralogy.org/, 2001; Accessed: 2024-07-23.

(17) Stoeck, T.; Bass, D.; Nebel, M.; Christen, R.; Jones, M. D. M.; Breiner, H.-W.; Richards, T. A. Multiple marker parallel tag environmental DNA sequencing reveals a highly complex eukaryotic community in marine anoxic water. *Molecular Ecology* **2010**, *19*, 21–31, DOI: 10.1111/j.1365-294X.2009.04480.x.

(18) Schloss, P. D.; Westcott, S. L.; Ryabin, T.; Hall, J. R.; Hartmann, M.; Hollister, E. B.; Lesniewski, R. A.; Oakley, B. B.; Parks, D. H.; Robinson, C. J.; Sahl, J. W.; Stres, B.; Thallinger, G. G.; Horn, D. J. V.; Weber, C. F. Introducing mothur: Open-Source, Platform-Independent, Community-Supported Software for Describing and Comparing Microbial Communities. *Appl. Environ. Microbiol.* **2009**, *75*, 7537–7541, DOI: doi:10.1128/AEM.01541-09.

(19) Kozich, J. J.; Westcott, S. L.; Baxter, N. T.; Highlander, S. K.; Schloss, P. D. Development of a Dual-Index Sequencing Strategy and Curation Pipeline for Analyzing Amplicon Sequence Data on the MiSeq Illumina Sequencing Platform. *Appl. Environ. Microbiol.* **2013**, *79*, 5112–5120, DOI: doi:10.1128/AEM.01043-13.

(20) White, T. J. Amplification and direct sequencing of fungal ribosomal RNA genes for phylogenetics. *PCR Protoc.* **1990**, 315–322.

(21) Conen, F.; Henne, S.; Morris, C. E.; Alewell, C. Atmospheric ice nucleators active ≥-12 degrees C can be quantified on PM10 filters. *Atmos. Meas. Tech.* **2012**, *5*, 321–327, DOI: 10.5194/amt-5-321-2012.

(22) Hartmann, M.; Adachi, K.; Eppers, O.; Haas, C.; Herber, A.; Holzinger, R.; Hunerbein, A.; Jakel, E.; Jentzsch, C.; van Pinxteren, M.; Wex, H.; Willmes, S.; Stratmann, F. Wintertime Airborne Measurements of Ice Nucleating Particles in the High Arctic: A Hint to a Marine, Biogenic Source for Ice Nucleating Particles. *Geophys. Res. Lett.* **2020**, *47*, DOI: 10.1029/2020gl087770.

(23) Vali, G. Quantitative Evaluation Of Experimental Results On Heterogeneous Freezing Nucleation Of Supercooled Liquids. *J. Atmos. Sci.* **1971**, *28*, 402–409, DOI: 10.1175/1520-0469(1971)028<0402:QEOERA>2.0.CO;2.

(24) Zeppenfeld, S.; van Pinxteren, M.; van Pinxteren, D.; Wex, H.; Berdalet, E.; Vaqué, D.; Dall'Osto, M.; Herrmann, H. Aerosol Marine Primary Carbohydrates and Atmospheric Transformation in the Western Antarctic Peninsula. *ACS Earth Space Chem.* **2021**, *5*, 1032–1047, DOI: 10.1021/acsearthspacechem.0c00351.

(25) Niedermeier, D.; Augustin-Bauditz, S.; Hartmann, S.; Wex, H.; Ignatius, K.; Stratmann, F. Can we define an asymptotic value for the ice active surface site density for heterogeneous ice nucleation? *J. Geophys. Res. Atmos.* **2015**, *120*, 5036–5046, DOI: 10.1002/2014jd022814.

(26) Niedermeier, D.; Shaw, R. A.; Hartmann, S.; Wex, H.; Clauss, T.; Voigtlaender, J.; Stratmann, F. Heterogeneous ice nucleation: exploring the transition from stochastic to singular freezing behavior. *Atmos. Chem. Phys.* **2011**, *11*, 8767–8775, DOI: 10.5194/acp-11-8767-2011.

(27) Hartmann, S.; Augustin, S.; Clauss, T.; Wex, H.; Santl-Temkiv, T.; Voigtlaender, J.; Niedermeier, D.; Stratmann, F. Immersion freezing of ice nucleation active protein complexes. *Atmos. Chem. Phys.* **2013**, *13*, 5751–5766, DOI: 10.5194/acp-13-5751-2013.

(28) Niedermeier, D.; Ervens, B.; Clauss, T.; Voigtlaender, J.; Wex, H.; Hartmann, S.; Stratmann, F. A computationally efficient description of heterogeneous freezing: A simplified version of the Soccer ball model. *Geophys. Res. Lett.* **2014**, *41*, 736–741, DOI: 10.1002/2013gl058684.

(29) Knackstedt, K. A.; Moffett, B. F.; Hartmann, S.; Wex, H.; Hill, T. C. J.; Glasgo, E. D.; Reitz, L. A.; Augustin-Bauditz, S.; Beall, B. F. N.; Bullerjahn, G. S.; Frohlich-Nowoisky, J.; Grawe, S.; Lubitz, J.; Stratmann, F.; McKay, R. M. L. Terrestrial Origin for Abundant Riverine Nanoscale Ice-Nucleating Particles. *Environ. Sci. Technol.* **2018**, *52*, 12358–12367, DOI: 10.1021/acs.est.8b03881.

(30) Zobrist, B.; Koop, T.; Luo, B. P.; Marcolli, C.; Peter, T. Heterogeneous ice nucleation rate coefficient of water droplets coated by a nonadecanol monolayer. *J. Phys. Chem. C* **2007**, *111*, 2149–2155, DOI: 10.1021/jp066080w.

(31) Vignati, E.; Wilson, J.; Stier, P. M7: An efficient size-resolved aerosol microphysics module for large-scale aerosol transport models. *J. Geophys. Res. Atmos.* **2004**, *109*, DOI: 10.1029/2003jd004485.

(32) van Noije, T.; Bergman, T.; Le Sager, P.; O'Donnell, D.; Makkonen, R.; Gonçalves-Ageitos, M.; Döscher, R.; Fladrich, U.; von Hardenberg, J.; Keskinen, J. P.; Korhonen, H.; Laakso, A.; Myriokefalitakis, S.; Ollinaho, P.; Pérez García-Pando, C.; Reerink, T.; Schrödner, R.; Wyser, K.; Yang, S. EC-Earth3-AerChem: a global climate model with interactive aerosols and atmospheric chemistry participating in CMIP6. *Geosci. Model Dev.* **2021**, *14*, 5637–5668, DOI: 10.5194/gmd-14-5637-2021.

(33) van Noije, T. P. C.; Le Sager, P.; Segers, A. J.; van Velthoven, P. F. J.; Krol, M. C.;

Hazeleger, W.; Williams, A. G.; Chambers, S. D. Simulation of tropospheric chemistry and aerosols with the climate model EC-Earth. *Geosci. Model Dev.* **2014**, *7*, 2435–2475, DOI: 10.5194/gmd-7-2435-2014.

(34) Bergman, T.; Makkonen, R.; Schrödner, R.; Swietlicki, E.; Phillips, V. T. J.; Le Sager, P.; van Noije, T. Description and evaluation of a secondary organic aerosol and new particle formation scheme within TM5-MP v1.2. *Geosci. Model Dev.* **2022**, *15*, 683–713, DOI: 10.5194/gmd-15-683-2022.

(35) Gliß, J.; Mortier, A.; Schulz, M.; Andrews, E.; Balkanski, Y.; Bauer, S. E.; Benedictow, A. M. K.; Bian, H.; Checa-Garcia, R.; Chin, M.; Ginoux, P.; Griesfeller, J. J.; Heckel, A.; Kipling, Z.; Kirkevåg, A.; Kokkola, H.; Laj, P.; Le Sager, P.; Lund, M. T.; Lund Myhre, C.; Matsui, H.; Myhre, G.; Neubauer, D.; van Noije, T.; North, P.; Olivié, D. J. L.; Rémy, S.; Sogacheva, L.; Takemura, T.; Tsigaridis, K.; Tsyro, S. G. AeroCom phase III multi-model evaluation of the aerosol life cycle and optical properties using ground- and space-based remote sensing as well as surface in situ observations. *Atmos. Chem. Phys.* **2021**, *21*, 87–128, DOI: 10.5194/acp-21-87-2021.

(36) Checa-Garcia, R.; Balkanski, Y.; Albani, S.; Bergman, T.; Carslaw, K.; Cozic, A.; Dearden, C.; Marticorena, B.; Michou, M.; van Noije, T.; Nabat, P.; O'Connor, F. M.; Olivié, D.; Prospero, J. M.; Le Sager, P.; Schulz, M.; Scott, C. Evaluation of natural aerosols in CRESCENDO Earth system models (ESMs): mineral dust. *Atmos. Chem. Phys.* **2021**, *21*, 10295–10335, DOI: 10.5194/acp-21-10295-2021.

(37) Vignati, E.; Facchini, M. C.; Rinaldi, M.; Scannell, C.; Ceburnis, D.; Sciare, J.; Kanakidou, M.; Myriokefalitakis, S.; Dentener, F.; O'Dowd, C. D. Global scale emission and distribution of sea-spray aerosol: Sea-salt and organic enrichment. *Atmos. Environ.* **2010**, *44*, 670–677, DOI: https://doi.org/10.1016/j.atmosenv.2009.11.013.

(38) McCluskey, C. S.; Hill, T. C. J.; Humphries, R. S.; Rauker, A. M.; Moreau, S.; Strut-

ton, P. G.; Chambers, S. D.; Williams, A. G.; McRobert, I.; Ward, J.; Keywood, M. D.; Harnwell, J.; Ponsonby, W.; Loh, Z. M.; Krummel, P. B.; Protat, A.; Kreidenweis, S. M.; DeMott, P. J. Observations of Ice Nucleating Particles Over Southern Ocean Waters. *Geophys. Res. Lett.* **2018**, *45*, 11989–11997, DOI: 10.1029/2018gl079981.

(39) Tatzelt, C.; Henning, S.; Welti, A.; Baccarini, A.; Hartmann, M.; Gysel-Beer, M.; van Pinxteren, M.; Modini, R. L.; Schmale, J.; Stratmann, F. Circum-Antarctic abundance and properties of CCN and INPs. *Atmos. Chem. Phys.* **2022**, *22*, 9721–9745, DOI: 10.5194/acp-22-9721-2022.

(40) McCluskey, C. S.; Ovadnevaite, J.; Rinaldi, M.; Atkinson, J.; Belosi, F.; Ceburnis, D.; Marullo, S.; Hill, T. C. J.; Lohmann, U.; Kanji, Z. A.; O'Dowd, C.; Kreidenweis, S. M.; DeMott, P. J. Marine and Terrestrial Organic Ice-Nucleating Particles in Pristine Marine to Continentally Influenced Northeast Atlantic Air Masses. *J. Geophys. Res. Atmos.* **2018**, *123*, 6196–6212, DOI: 10.1029/2017jd028033.

(41) DeMott, P.; Moore, K. CAPRICORN R/V Investigator Ice Spectrometer Measurements. Version 1.0. UCAR/NCAR - Earth Observing Laboratory. 2022.

(42) DeMott, P.; Hill, T. C. J.; McFarquhar, G. MARCUS Ice Nucleating Particle Measurements. 2020.

(43) Hill, T. C. J.; DeMott, P. Ice nucleating particle concentrations at Macquarie Island, 2017/8. 2020.

(44) DeMott, P. J.; Hill, T. C. *ACAPEX – Ship-Based Ice Nuclei Collections Field Campaign Report*; Report, 2016.

(45) Bumke, K. *The expedition of the research vessel "Polarstern" to the Antarctic in 2012 (ANT-XXVIII/5)*; Report, 2012; DOI: 10.2312/BzPM_0654_2012.

(46) Knust, E.; Lochte, K. *The Expeditions PS95.1 and PS95.2 of the Research Vessel POLARSTERN to the Atlantic Ocean in 2015*; Report, 2016; DOI: 10.2312/BzPM_0702_2016.

(47) Hartmann, M.; Gong, X.; Welti, A.; Stratmann, F. Shipborne Ice Nucleating Particle (INP) measurements in the Arctic during PS106.1 and PS106.2. 2020.
